# Origin of Room Temperature Methanol Synthesis over Hcp‐PdMo

**DOI:** 10.1002/anie.202505634

**Published:** 2025-06-03

**Authors:** Feilong Xing, Ruopeng Wang, Shiyao Wang, Hironobu Sugiyama, Chenyang Zhu, Masayoshi Miyazaki, Hideo Hosono, Masaaki Kitano

**Affiliations:** ^1^ MDX Research Center for Element Strategy Institute of Integrated Research Institute of Science Tokyo 4259 Nagatsuta, Midori‐ku Yokohama 226–8503 Japan; ^2^ Advanced Institute for Materials Research (WPI‐AIMR) Tohoku University Sendai 980–8577 Japan; ^3^ International Center for Materials Nanoarchitectonics (WPI‐MANA) National Institute for Materials Science (NIMS) Tsukuba Ibaraki 305‐0044 Japan

**Keywords:** CO_2_ hydrogenation, Heterogeneous catalyst, Methanol, PdMo intermetallic, Room temperature

## Abstract

The hydrogenation of CO_2_ to methanol has garnered significant interest with respect to the reduction of carbon emissions; however, the high temperatures and pressures typically required diminish the benefits of this approach. In this study, we report a hexagonal close‐packed (hcp)‐PdMo catalyst that exhibits the highest room temperature catalytic activity for CO_2_ hydrogenation to methanol among reported catalysts, with 100% methanol selectivity and without any signs of deactivation. Structural analyses, which included various in situ and ex situ X‐ray techniques and infrared spectroscopy, have revealed that Mo and Pd serve as the active sites for CO_2_ adsorption and H_2_ dissociation, respectively. The CO_2_ hydrogenation reaction is facilitated at room temperature on the surface of the hcp‐PdMo intermetallic catalyst, where the adjacently arranged Pd and Mo sites play important roles in methanol synthesis. Mechanistic studies, combined with density functional theory (DFT) calculations, have demonstrated that the reaction proceeds via a reverse water‐gas shift and subsequent CO hydrogenation (R&C) pathway though a Pd‐assisted Mo redox mechanism at room temperature. These findings not only reveal the origin of the reaction mechanism for methanol synthesis but also open a new direction for the design of highly efficient catalysts that function under mild conditions.

## Graphical abstract

The hexagonal close‐packed PdMo (hcp‐PdMo) intermetallic catalyst exhibits the room temperature catalytic activity for CO_2_ hydrogenation to methanol, where the adjacently arranged Pd and Mo sites play important roles in methanol synthesis. Mechanism study revealed that the reaction proceeds via a reverse water‐gas shift and subsequent CO hydrogenation (R&C) pathway though a Pd‐assisted Mo redox mechanism at room temperature.

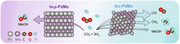

## Introduction

Methanol is one of the most crucial raw materials in modern society and serves as an intermediate for fine chemical synthesis and as a hydrogen energy carrier for transportation.^[^ ^1^, ^2^, ^3^, ^4^ ^]^ Historically, methanol has been primarily produced from syngas, which is generated by reforming or oxidizing fossil fuels at high temperatures (typically over 700 °C). However, current global environmental challenges, driven by significant energy consumption and the non‐reusability of fossil resources, have necessitated the exploration of alternative pathways for methanol synthesis. The conversion of CO_2_ to methanol has gained considerable attention due to the contribution to value‐added chemical production with a carbon capture and utilization (CCU) strategy for a future carbon‐neutral society. Over the past decades, the modification of commercial catalysts (Cu/ZnO/Al_2_O_3_)^[^ ^5^, ^6^, ^7^, ^8^, ^9^ ^]^ and the discovery of Cu‐free catalysts, such as bimetallic^[^ ^10^, ^11^, ^12^, ^13^ ^]^ and metal oxide catalysts,^[^ ^14^, ^15^, ^16^, ^17^ ^]^ have been extensively advanced with an aim of enhancing methanol selectivity and catalyst stability. Despite the exothermic nature of CO_2_ hydrogenation to methanol, few studies^[^ ^18^, ^19^, ^20^ ^]^ have reported the successful synthesis of methanol at low temperatures (≤100 °C) due to the high activation energy of the intrinsically stable CO_2_ molecules and the subsequent multi‐hydrogenation steps. Given the scientific significance and industrial potential of environmentally friendly and energy‐saving applications, there is an urgent need to develop highly efficient and valuable catalysts that can operate under mild conditions.

Elucidation of the origin of active sites in the catalyst structure to understand the reaction mechanism is of vital importance to accelerate the development of low‐temperature methanol synthesis catalysts. Mo‐based catalysts^[^ ^21^, ^22^, ^23^, ^24^, ^25^ ^]^ have previously attracted significant interest in various CO_2_ hydrogenation reactions due to promising results. The in‐plane sulfur vacancies of MoS_2_ nanosheets^[^ ^19^ ^]^ were reported to serve as active sites for both CO_2_ dissociation and H_2_ activation. While another study reported that Mo_3_S_4_ clusters^[^ ^26^ ^]^ act as active centers, with CO_2_ chemisorbed on the same Mo site via both carbon and oxygen, followed by three steps of hydrogenation from CO* to CH_3_O*. On the other hand, it is supposed that Mo sites oxidized by CO_2_ adsorption would be difficult to reduce at low reaction temperatures, especially room temperature, which would hinder continuous methanol production. We recently reported an air‐stable hcp‐PdMo intermetallic^[^ ^20^ ^]^ as a highly efficient catalyst for CO_2_ hydrogenation to methanol. The hcp‐PdMo produced methanol continuously even at room temperature and 0.9 MPa. However, the reasons for the structural formation and stabilization of the hcp‐PdMo intermetallic catalyst, the role of individual elements in the structure, and the specific morphology and changes during the reaction remain unclear.

Herein, we investigate the different crystal structures of Pd‐Mo catalysts through a combination of X‐ray diffraction (XRD) measurements, spectroscopic techniques, and theoretical analysis using density functional theory (DFT) calculations to elaborate on the structure‐performance relationship and provide a detailed understanding of the reaction mechanism. The results revealed that the ordered layer structure of the hcp‐PdMo intermetallic is a crucial factor for the continuous production of methanol at low temperature, and that increasing the surface area of the hcp‐PdMo can significantly increase the methanol synthesis rate.

## Results and Discussion

### Catalyst Structure and Catalytic Performance

**Figure 1:**
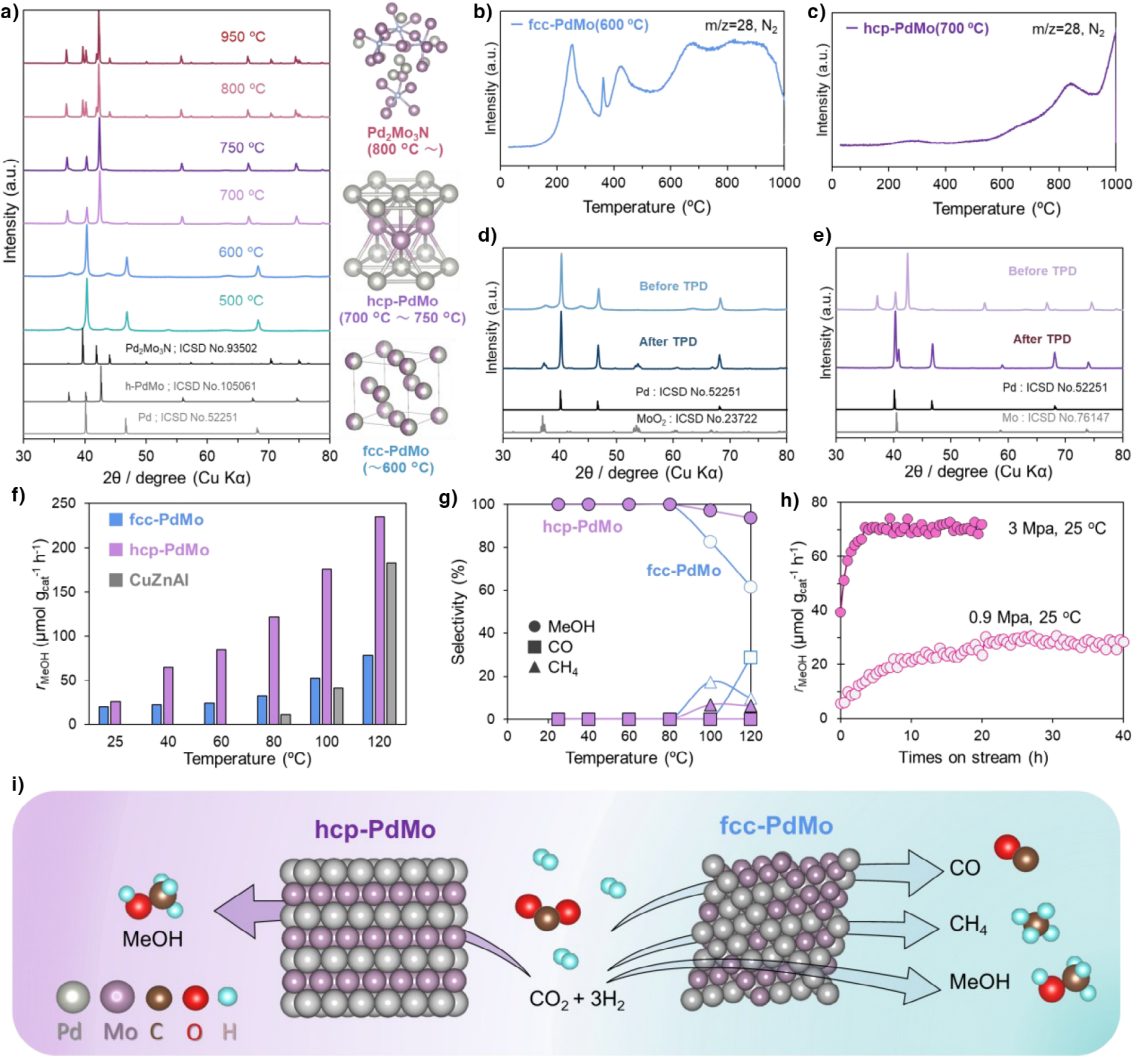
Structure‐dependent activity of Pd‐Mo catalysts. a) XRD patterns and structures for catalysts calcined at different temperatures. TPD measurements of the catalyst calcined at b) 600 °C and c) 700 °C. XRD patterns for the catalysts calcined at d) 600 °C and e) 700 °C before and after TPD measurement. f) CH_3_OH formation rate and g) product selectivity from 25°C to 120 °C over the PdMo catalysts and the reference Cu/ZnO/Al_2_O_3_ (CuZnAl) commercial catalyst. Reaction conditions: 0.1 g catalyst, CO_2_: H_2_: Ar = 10:30:10 mL min^−1^, 0.9 MPa. h) long‐term stability test of HSA hcp‐PdMo catalyst for methanol synthesis activity at room‐temperature under pressurized conditions. i) Schematic illustration of the differences in selectivity between the hcp‐PdMo and fcc‐PdMo catalysts.

A series of Pd‐Mo catalysts was first prepared under various ammonia nitridation temperatures to investigate the structure‐dependent activity of the hcp‐PdMo catalysts. Figure 1a shows XRD patterns for the resultant materials. The face‐centered cubic (fcc)‐Pd phase was identified as the main phase below 600 °C, and MoO_2_ and Mo_3_N_2_ were detected as impurity phases in the catalysts prepared at 500 °C and 600 °C, respectively. Scanning electron microscopy‐energy dispersive X‐ray spectroscopy (SEM‐EDX) results (Figure S1) indicated that Pd and Mo were uniformly dispersed in the catalyst calcined at 600 °C. These results suggest that a PdMo alloy with an fcc structure is formed below 600 °C (denoted as fcc‐PdMo). The hcp‐PdMo phase then starts to form above 700 °C (denoted as hcp‐PdMo) and remains stable at 750 °C. The phase diagram for Pd and Mo indicates that hcp‐PdMo is generally a high‐temperature phase that exists only at temperatures above 1300 °C; therefore, the hcp‐PdMo phase easily segregates to form Pd and Mo below this temperature. This is the first time that the hcp‐PdMo structure has been demonstrated at such a low temperature (≤750 °C), which is attributed to the presence of anions that stabilize the metastable hcp‐PdMo phase. Except for the peaks associated with (002) planes (enlarged XRD pattern in Figure S2), all diffraction peaks in the hcp‐PdMo intermetallic structure were shifted to lower angles, which indicates that lattice expansion occurs more in the in‐plane direction than in the vertical direction. However, when the temperature exceeds 800 °C, a new ternary nitride phase of Pd_2_Mo_3_N begins to form and coexists with the hcp‐PdMo structure. Pd_2_Mo_3_N has been reported as a stable molybdenum bimetallic interstitial nitride phase that can be synthesized by ammonolysis processes at 1000 °C.^[^ ^27^ ^]^ In this study, the formation of both metastable fcc‐PdMo and hcp‐PdMo phases may be attributed to the anion stabilization effect. Therefore, temperature‐programmed desorption (TPD) measurements were conducted to identify the anion species. Nitrogen desorption from fcc‐PdMo was initiated in the low‐temperature range around 200 °C and continued up to 1000 °C (Figure 1b). In contrast, nitrogen desorption in hcp‐PdMo (Figure 1c) commenced gradually at temperatures above 600 °C, with desorption rates increasing progressively with temperature. This behavior indicates that nitrogen within the hcp‐PdMo intermetallic compound is more resistant to desorption and likely forms highly stable sites within the intermetallic structure. After TPD measurements, distinct phases of Pd and MoO_2_ were observed in the fcc‐PdMo phase (Figure 1d), whereas separate phases of Pd and Mo were identified in the hcp‐PdMo phase (Figure 1e). These results indicate that anion desorption triggers the aggregation of Pd in PdMo under Ar flow, which leads to the formation of metallic Pd. Numerous oxygen ions are present in the fcc‐PdMo lattice or as an amorphous oxide, which could bond with molybdenum to form MoO_2_ after the desorption of N anions. Based on these observations, it can be concluded that both fcc‐PdMo and hcp‐PdMo represent metastable phases, wherein the presence of anions within the lattice plays a critical role in stabilization of the structure at lower temperatures. This suggests that the incorporation of anions is essential to maintain the structural integrity of these phases under mild conditions.

Figure S3 shows the activity of these catalysts for methanol synthesis at 0.1 MPa. The PdMo catalyst prepared at 700 °C exhibited similar activity to that reported in our previous study,^[^ ^20^ ^]^ which confirmed the good reproducibility of the catalytic activity. The fcc‐PdMo catalyst synthesized at 600 °C exhibited similar activity to the hcp‐PdMo catalysts in the low‐temperature region, with no significant change in the apparent activation energy (Ea) for CH_3_OH formation (Figure S5). This result suggests that both the random adjacency of Pd and Mo sites in the solid‐solution alloy (fcc‐PdMo) and the regularly ordered arrangement in the hexagonal close‐packed intermetallic compound structure (hcp‐PdMo) have a significant impact on the methanol synthesis activity. However, the fcc‐PdMo exhibited a lower Ea for CO formation than the hcp‐PdMo catalyst (Figure S5), which led to a higher tendency for the generation of byproducts such as CO at low temperature (Figures S4b and S4c). The Pd and Mo atoms in fcc‐PdMo are randomly arranged, which leads to the formation of both Pd‐Mo adjacent sites and Pd aggregation sites. The former contributes to CH_3_OH formation in a similar manner to the hcp‐PdMo surface, whereas the latter accelerates the reverse water‐gas shift (RWGS); Pd_3_ clusters predominantly produce CO,^[^ ^28^, ^29^, ^30^ ^]^ which results in lower CH_3_OH selectivity. This indicates that the ordered layer structure of hcp‐PdMo suppresses side reactions more effectively under mild conditions. On the other hand, the catalytic activity significantly decreased when the catalyst was calcined above 800 °C, and those calcined at 950 °C did not produce methanol, which may be due to the formation of the inactive Pd_2_Mo_3_N phase or the low surface area of the Pd_2_Mo_3_N phase (Table S1). The CO_2_ hydrogenation reaction was further conducted under pressurized conditions to evaluate the catalytic activities of the fcc‐PdMo and hcp‐PdMo catalysts at low temperatures (Figure S6). As expected, both of the methanol synthesis activity and selectivity of the PdMo catalyst was much improved at 0.9 MPa. Notably, methanol production was observed even at room temperature (Figure 1f). On the other hand, the commercial Cu/ZnO/Al_2_O_3_ catalyst, as the benchmark catalyst for this reaction, exhibited a methanol production rate comparable to that of hcp‐PdMo catalyst at 120 °C, but had a much lower activity below 100 °C. Meanwhile, the fcc‐PdMo catalyst exhibits much lower activity and selectivity toward methanol than hcp‐PdMo (Figure 1g). Its random atomic arrangement of the former not only limits the methanol production rate but also leads to the formation of significant amounts of by‐products such as CH_4_ and CO. To demonstrate the unique catalytic performance of the PdMo catalyst and exclude the possibility of background reactivity, we conducted blank reactor tests under various reaction conditions, and found no methanol formation peaks at either room temperature or high temperatures under pressurized conditions, even after prolonged reaction times (Figure S7). It should be noted that the catalytic performance of the hcp‐PdMo catalyst synthesized at 750 °C was further enhanced by the increase in the surface area (HSA hcp‐PdMo, Table S1) when polyvinylpyrrolidone was used as a chelating agent instead of citric acid. The resultant HSA hcp‐PdMo catalyst exhibited a CH_3_OH formation rate of 25.6 µmol·g^−1^·h^−1^ at room temperature under 0.9 MPa pressure (Figure 1h), which was approximately three times higher than that for the previous state‐of‐the‐art hcp‐PdMo catalyst.^[^ ^20^ ^]^ Long‐term stability tests were conducted under both 0.9  and 3 MPa conditions. The initial increase in the CH_3_OH formation rate is likely due to the required activation time for the reaction to reach steady‐state equilibrium at room temperature. The HSA hcp‐PdMo catalyst maintained continuous methanol production without deactivation under pressurized conditions. As summarized in Table S2, most of the reported catalysts that exhibit high methanol selectivity typically require elevated reaction temperatures (≥200 °C) to achieve measurable activity. Even though, these systems often suffer from low CO_2_ conversion rates (usually <2%) under high WHSV conditions (>12 000 mL g^−1^ h^−1^), highlighting a critical trade‐off between activity and selectivity. Inspiringly, our HSA hcp‐PdMo catalyst enables nearly 100% methanol selectivity even at room temperature (25 °C), although the conversion remains low due to kinetic limitations. Importantly, the methanol productivity of HSA hcp‐PdMo reaches up to ∼72.6. µmol·g^−1^·h^−1^ at 25 °C and 3 MPa, which is notably higher than previously reported catalysts under similar low‐temperature conditions. This result not only highlights the unique potential of the PdMo catalyst for selective methanol synthesis under mild conditions, but also opens new avenues for designing active low‐temperature hydrogenation catalysts.

**Figure 2:**
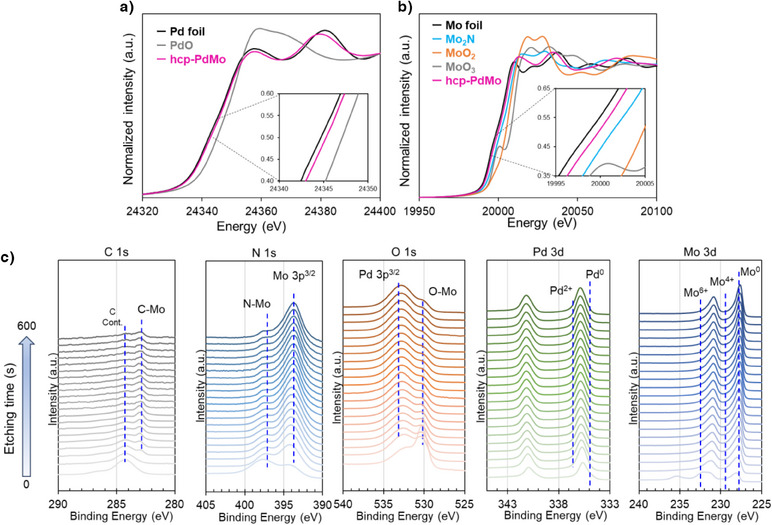
Structural analysis of the hcp‐PdMo catalyst (a) Pd K and (b) Mo K‐edge XANES spectra of the hcp‐PdMo catalyst and reference compounds. c) Time‐dependent XPS C 1s, N 1s, O 1s, Pd 3d, and Mo 3d spectra with Ar^+^ sputtering for the hcp‐PdMo catalyst, respectively.

A more comprehensive structural analysis of the hcp‐PdMo catalyst is necessary to investigate the structure‐performance relationship. The hcp‐PdMo crystal structure has alternating stacked layers of Pd and Mo (Figure 1a), and high‐angle annular dark‐field‐scanning transmission electron microscopy (HAADF‐STEM) images showed that Pd and Mo are aligned in a regularly ordered arrangement.^[^ ^20^ ^]^ The shift of the (002) peak is smaller than those for peaks associated with other planes (Figure S2), which suggests a lower likelihood of anions being present between these layers and indicates that shifts occur more in the in‐plane direction. To verify the location of the anions and the metallic electronic states of the hcp‐PdMo catalysts, X‐ray absorption fine‐structure spectroscopy (XAFS) and X‐ray photoelectron spectroscopy (XPS) measurements were performed. The Pd K‐edge and Mo K‐edge X ray absorption near‐edge spectra (XANES) of the hcp‐PdMo catalysts were close to those of the corresponding metal foils (Figure 2a,b), which indicates that these metals were mostly reduced to a zero‐valent state. The enlarged spectra (inset of Figure 2a,b) of both the Pd and Mo K‐edges exhibit a slight shift toward higher energy due to electron deficiency, which can be attributed to the interaction with highly electronegative anions present in the hcp‐PdMo lattice. The higher anionic affinity of Mo than Pd results in Mo being surrounded by more anions, which renders it more electron‐deficient. This electron deficiency further explains the stabilization of Mo in the hcp‐PdMo structure. Depth‐profiling elemental analysis with the XPS technique and Ar^+^ sputtering was conducted to determine whether the anion species are more abundant on the surface or in the interior of the catalyst. Figure 2c presents summarized data of the relationship between the Ar^+^ sputtering time and the XPS spectra. The electronic state of Pd is 335.6 eV, which is between Pd and Pd^2+^, and the shift is attributed to a change in the local environment due to alloying effects.^[^ ^30^, ^31^, ^32^ ^]^ In the initial stage, the O 1s peak at 530.2 eV rapidly decreases accompanied by the disappearance of the Mo^6+^ peak, which is likely due to contamination of the catalyst during transfer. Even after etching for 600 s by Ar^+^ sputtering, the presence of N, O, and a small amount of C were confirmed, which suggests that these anions were incorporated into the bulk of the catalyst, and likely entered the lattice and contributed to the stabilization of hcp‐PdMo. Further detailed localized information was analyzed through the extended‐XAFS (EXAFS) and corresponding Fourier transformed spectra, as shown in Figure S8. The Pd K‐edge spectra showed that the coordination environment of Pd in the hcp‐PdMo catalyst is similar to that of the metal state (Figures S8a and S8b), primarily bonded with metal atoms (Pd and/or Mo). This indicates that there is negligible presence of anions in the coordination environment of Pd. In contrast, the Mo K‐edge EXAFS spectrum of the hcp‐PdMo catalyst is significantly different from that of Mo foil (Figures S8c and S8d). In addition to the peaks that correspond to Mo─Mo(Pd) bonds with bond lengths exceeding 2 Å, peaks associated with Mo─N and Mo─O bonds with lengths shorter than 2 Å were also detected. This suggests that the anions are primarily present within the Mo─Mo plane of the stacked layer structure rather than in the Pd─Pd plane. The small amplitude of vibrations that originate from Mo─Mo bonds implies that Mo in the hcp‐PdMo catalyst is irregularly bonded to anions and possesses a coordination environment that is significantly different from that for metallic Mo. Overall, the observed electron deficiency of both Pd and Mo is attributed to electron donation to anions present within the hcp‐PdMo catalyst; Mo is more electron‐deficient than Pd and more light elements are present in the surrounding coordination environment, which infers that anions are more prevalent around Mo than Pd.

The catalytic performance of the hcp‐PdMo and Pd‐based catalysts was compared to demonstrate the advantages of the hcp‐PdMo structure in terms of methanol synthesis activity. Figure S9a presents XRD patterns for the Pd‐based catalysts, where both the 5 wt% Pd/Mo_2_N and Pd/MoO_2_ catalysts show Pd peaks, which confirms the presence of Pd metal particles on their respective supports. A 5 wt% hcp‐PdMo/Mo_2_N catalyst was also synthesized for comparison, which exhibited comparable catalytic performance to a 55 wt% single phase hcp‐PdMo catalyst (Figure S9b) and with no significant difference in activation energy. However, the methanol formation rate was significantly decreased for the 5 wt% Pd/Mo_2_N catalyst, and no methanol synthesis activity was observed for the 5 wt% Pd/MoO_2_ catalyst. Moreover, the activation energy for the Pd/Mo_2_N catalyst for methanol synthesis was 77.9 kJ mol^−1^, which was higher than that for the hcp‐PdMo catalyst and indicated that hcp‐PdMo can produce methanol at lower temperatures and with lower activation energy than conventional Mo‐nitride and Mo‐oxide‐supported monometallic Pd catalysts. The preferential crystallographic orientation of the structures can influence the catalytic performance, particularly in the layered hcp‐PdMo structure where identification of the active surface is crucial. To confirm our hypothesis that the (101) planes perpendicular to the layer stacking direction function as the active sites, two catalysts with different orientation preferences were prepared, and the textural coefficient^[^ ^33^ ^]^ was calculated from the XRD peaks (Figure S10a), one with preferential exposure of the (101) plane and the other with equivalent exposure of the (101) and (002) planes. The results showed that the catalyst with preferential exposure of the (101) plane (regularly ordered Pd‐Mo arrangement at the exposed surface) exhibited a higher methanol production rate, while the catalyst without preferential exposure of the (101) plane (mostly Pd or Mo at the exposed surface) favored CO production (Figures S10d and S10e). These results illustrate the unique structure of hcp‐PdMo, wherein the atomic‐scale adjacency of Pd and Mo plays a crucial role in the hydrogenation of CO_2_ to methanol under mild conditions.

### Catalytic Mechanism

**Figure 3:**
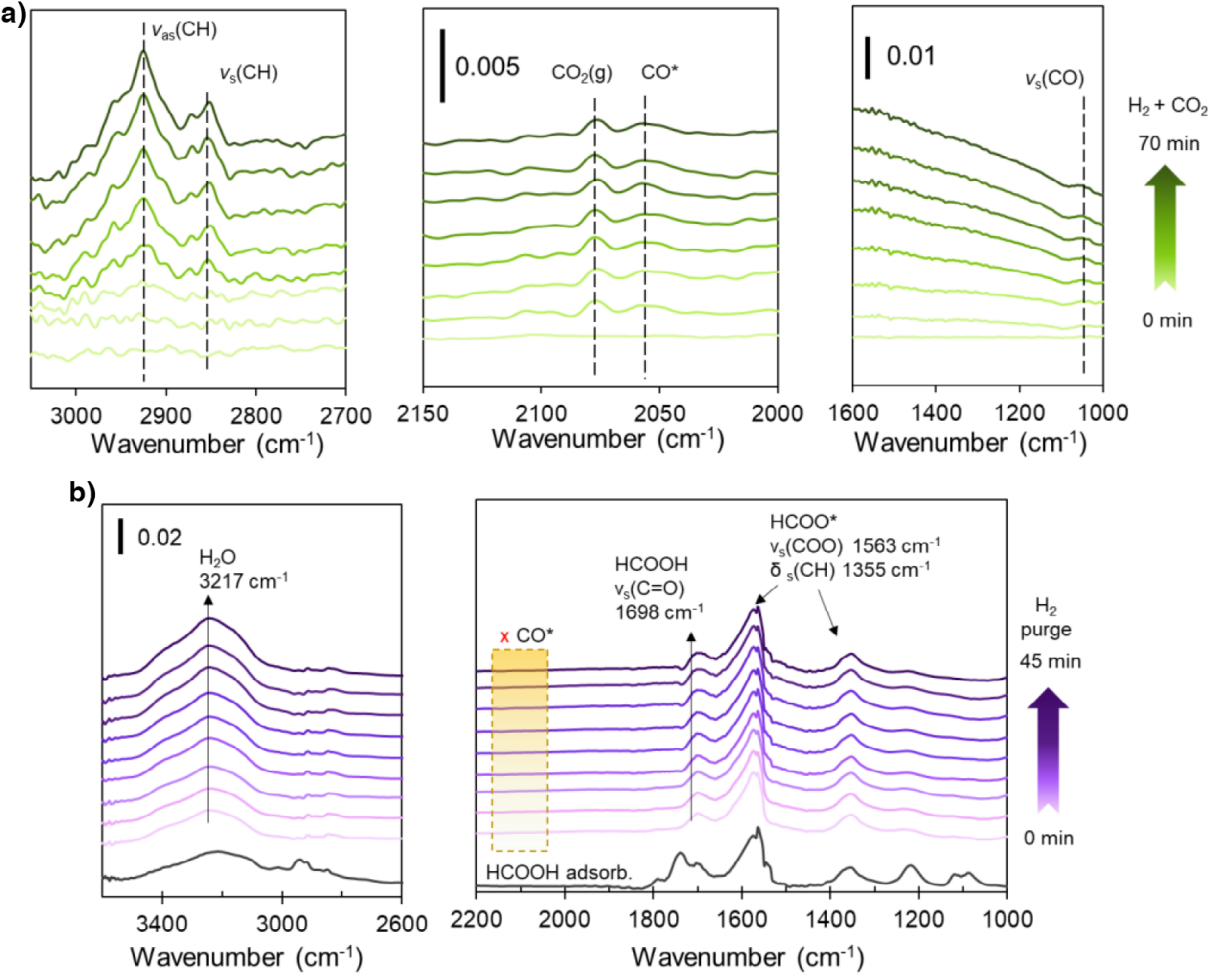
DRIFT spectra of the reduced 5 wt% hcp‐PdMo/Mo_2_N catalyst at room temperature. a) CO_2_ hydrogenation and b) adsorbed formic acid hydrogenation.

To investigate the factors that contribute to the low‐temperature activity of the hcp‐PdMo catalyst, in situ diffuse reflectance infrared Fourier transform (DRIFT) spectroscopy measurements were performed under methanol synthesis conditions. The catalyst used in the measurements was 5wt% hcp‐PdMo/Mo_2_N, which possesses a higher surface area than the single‐phase hcp‐PdMo catalyst. As described in our previous work,^[^ ^20^ ^]^ the reaction mechanism for methanol synthesis for the hcp‐PdMo catalysts more likely involves an RWGS and subsequent CO hydrogenation (R&C) pathway. After introducing a CO_2_ + H_2_ gas mixture into PdMo catalyst at room temperature, two adsorption peaks were observed at 2076 and 2054 cm^−1^ (Figure 3a, middle). These peaks are likely due to gas‐phase CO_2_^[^ ^34^, ^35^ ^]^ since both peaks are observed for SiC (Figure S13a). However, the lower‐wavenumber bands differ from those of SiC in both shape/intensity and position, which may be caused by the contribution of CO adsorption peak. A sharp CO adsorption peak was observed at 2056 cm^−1^ when pure CO gas was introduced into PdMo catalyst (Figure S13b). To further confirm whether the observed CO peak is generated from CO_2_ activation, complementary CO_2_‐TPSR experiments were performed. As shown in Figure S13c, CO_2_ consumption and CO formation were observed, in contrast to no CO_2_ consumption and CO production for blank test. Moreover, as shown in Figure 3a, no signals corresponding to formate species were observed in the range of 1300–1600 cm^−1^ effectively ruling out the possibility of methanol formation via formate hydrogenation. Peaks derived from C─H symmetric stretching (2852 cm^−1^), C─H asymmetric stretching (2925 cm^−1^), and C─O stretching vibration of CH_3_O* species then gradually appeared after 30 min (Figure 3a, left). These results suggested that hcp‐PdMo catalyst may produce methanol from CO_2_ via a CO intermediate formation. The wavenumbers for the CO* species were shifted to the lower regions than those of the Pd and Mo‐based catalysts (Table S3), which indicates that the adsorbed CO* species exist in a more activated state due to electron donation from Mo. DFT calculations (Figure S11) revealed that the bond length of CO adsorbed on the Pd (111) surface is approximately 1.148 Å, which is slightly longer than that of gas‐phase CO (1.128 Å). Interestingly, CO preferentially adsorbs on the Mo site of the PdMo catalyst, further elongating its bond length to 1.191 Å. This bond elongation corresponds to the C–O bond weakening, leading to a red‐shift in the CO vibrational frequency, which is consistent with our DRIFT experimental results. Similarly, the adsorbed CH_3_O* species exhibited bands at wavenumbers closer to those for MoS_2_ (2846, 2915 cm^−1^)^[^ ^19^ ^]^ than to Pd (2860, 2960 cm^−1^), which suggests that the CH_3_O* species also adsorb on Mo sites in the hcp‐PdMo catalyst. DFT calculations were conducted to calculate the CO_2_ adsorption energy on the Pd (111) and PdMo (010) surfaces, along with a Bader charge analysis (Figure S12). Metallic Pd typically has high efficiency for the homolytic dissociation of hydrogen, but is less effective in the activation of CO_2_.^[^ ^36^, ^37^ ^]^ In the case of hcp‐PdMo, CO_2_ is strongly adsorbed on the Mo bridge sites of hcp‐PdMo with a large adsorption energy (E_ads_ = −0.7 eV) due to the better electrophilic properties of Mo atoms, which results in CO_2_ bending upon adsorption. A decrease in the Mo electron density accompanied an increase in the electron density of the carbon atom in CO_2_, which indicates electron transfer from Mo to CO_2_, leading to a more electron‐rich carbon state. The bent geometry of the CO_2_ adsorption state formed is due to electron filling of the lowest unoccupied molecular orbital, which lowers the energy state of CO_2_ adsorption and weakens the C‐O bond. This suggests that Mo sites in the hcp‐PdMo catalysts are effective in the activation of CO_2_ to CO with a low activation barrier than that for the Pd catalysts. Formic acid adsorption and hydrogenation on the hcp‐PdMo catalyst was also performed to investigate the possibility of the formation of formate (HCOO*) intermediates from CO_2_ hydrogenation. The experiment was conducted by introducing formic acid, evacuation to confirm the adsorption of formic acid, and then hydrogenation by purging H_2_. After the introduction of formic acid and evacuation (Figure S14), peaks due to molecularly adsorbed HCOOH (1698 and 1087 cm^−1^) and HCOO* species (around 1563 and 1355 cm^−1^) on the catalyst surface were confirmed. In addition, bonds for adsorbed formaldehyde (CH_2_O) species at 1739, 1220, and 1117 cm^−1^ were also observed because of the low energy barrier for the first C–O bond dissociation (HCOOH + M → CH_2_O + M–O). However, even after continued H_2_ purging, no CO* and CH_3_O* species were observed (Figure 3b), suggesting that the decomposition of HCOO* to CO* and further hydrogenation to CH_3_O* is not a favored pathway. It should be noted that the formaldehyde species generated over the hcp‐PdMo catalysts was also not decomposed to CO, which is typically formed on metallic catalyst surfaces (Pt, Au, and Rh), even at room temperature.^[^ ^38^ ^]^ These results offer further support that CO is primarily generated via direct CO_2_ dissociation and then hydrogenation to produce CH_3_O* via the R&C mechanism.

**Figure 4:**
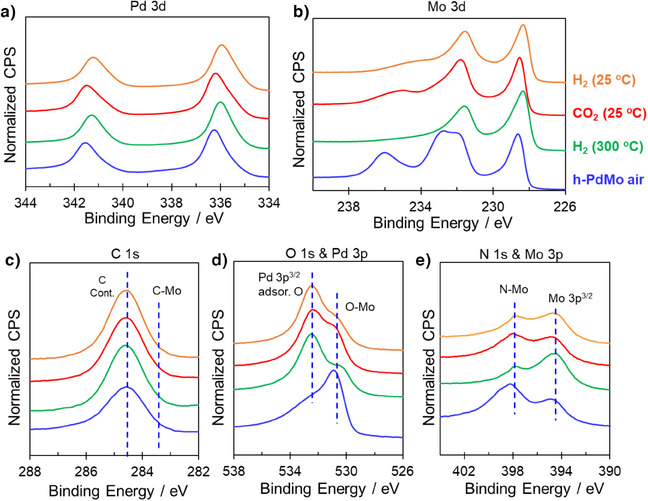
Ex situ XPS of the hcp‐PdMo catalyst. a) Pd 3d, b) Mo 3d, c) C 1s, d) O 1s/Pd 3p, and e) N 1s/Mo 3p spectra. Air exposed hcp‐PdMo catalysts (blue) and H_2_ treatment at 300 °C (green). The hcp‐PdMo catalysts H₂‐treated at 300 °C were treated with CO_2_ at 25 °C (red) and then further treated with H_2_ at 25 °C (orange).

**Figure 5:**
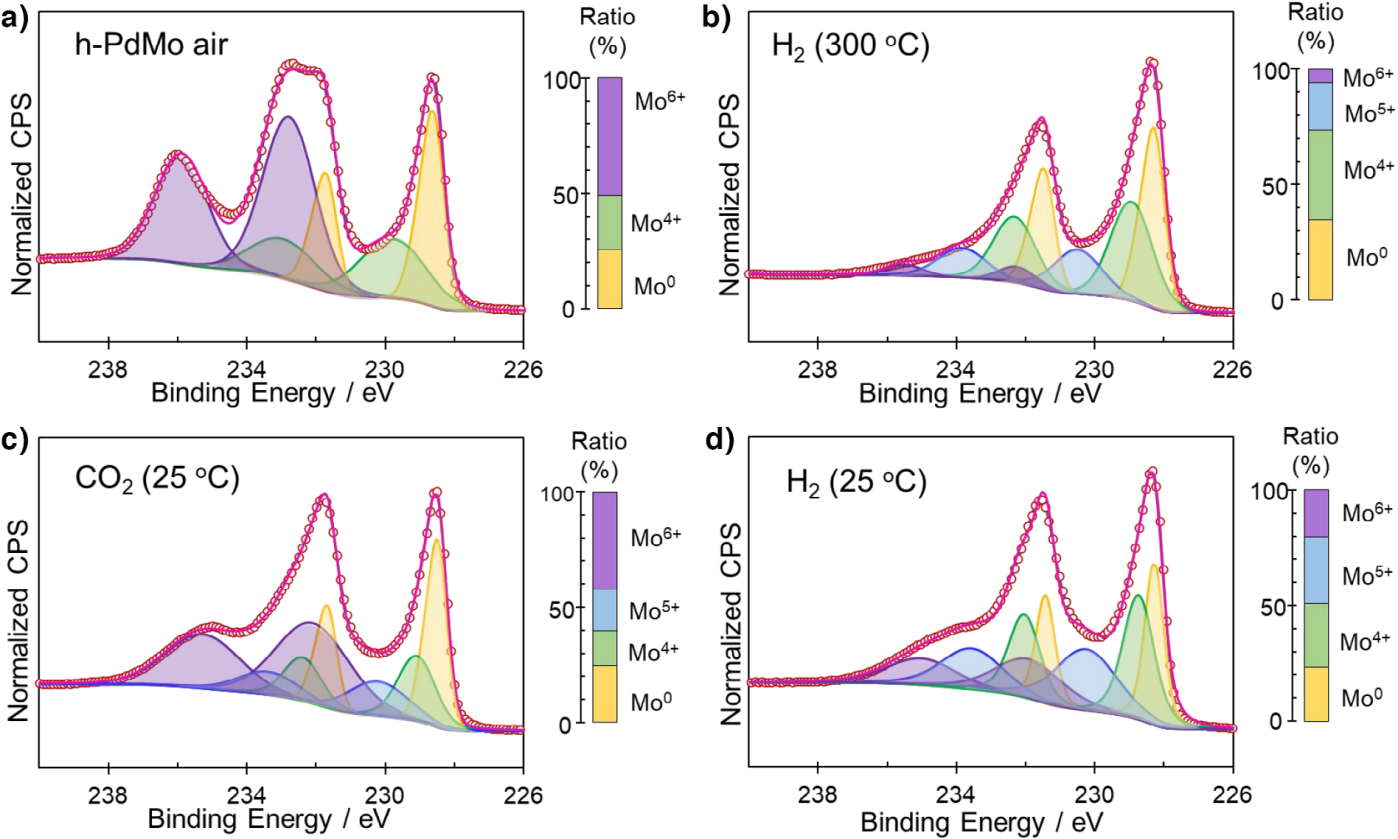
XPS Mo 3d peak fitting spectra and peak ratios; the Mo 3d peak in the hcp‐PdMo catalyst was deconvoluted into four valence states. a) Air‐exposed hcp‐PdMo catalyst and b) H_2_ treatment at 300 °C. c) The H₂‐prereduced hcp‐PdMo catalyst was treated with CO_2_ at 25 °C and then (d) further treated with H_2_ at 25 °C.

Verification of the dynamic changes in a structure during the reaction is essential for the rational design of a catalyst.^[^ ^24^ ^]^ Therefore, to confirm the local structure and surface state of the hcp‐PdMo catalysts, ex situ XAS and XPS measurements were conducted after treatment with CO_2_ and H_2_ gases. Figure S15 shows Pd‐K and Mo‐K edge XANES and EXAFS spectra of the hcp‐PdMo catalyst, which revealed no significant difference in the spectral shape after treatments with CO_2_, H_2_, and a CO_2 _+ H_2_ mixture. This indicates that the bulk structure was maintained during the catalytic reaction. Such a stable structure of hcp‐PdMo contributed to the continuous synthesis of methanol at room temperature. Meanwhile, when CO_2_ was introduced into the H_2_‐prereduced hcp‐PdMo catalyst, the electronic state of Mo in the XANES spectrum (Figure S15b) was slightly oxidized at room temperature, whereas the electronic state of Pd remained almost unchanged (Figure S15a). This is consistent with the in situ DRIFT measurements, which revealed that CO_2_ dissociates into CO* and O* on Mo sites, with the dissociated O* species oxidizing Mo. The reversible change of the Mo electronic states during the CO_2_ hydrogenation reaction is similar to that observed at 180 °C in the few‐layered sulfur vacancy‐rich MoS_2_ (FL‐MoS_2_) nanosheet catalyst.^[^ ^19^ ^]^ However, unlike the irreversible S vacancies in FL‐MoS_2_ caused by oxidation, the Mo sites in the hcp‐PdMo catalyst can be reduced even at room temperature under a H_2_ gas purge (Figure S15b). This reduction capability contributes to the superior stability and sustained catalytic activity of the hcp‐PdMo catalyst under room temperature conditions. The reversible redox cycle of the surface Mo sites on the hcp‐PdMo catalyst was further confirmed by ex situ XPS measurements with the same treatment conditions, as summarized in Figure 4. The pre‐reduced Pd exhibited characteristic Pd 3d peaks that correspond to a metallic state (Figure 4a). After CO_2_ treatment, a slight shift to higher binding energy (Pd^δ+^) was observed, which suggests the possibility of interaction between O* species and Pd when high‐coverage O* species are present. This partially oxidized Pd^δ+^ state was fully restored to the metallic state following H_2_ treatment. Additionally, there were many Mo^6+^ sites on the surface of the hcp‐PdMo catalyst stored in an air atmosphere (Figure 4b and Table S4); however, these oxidized Mo^6+^ were reduced to lower valence states such as Mo^0^, Mo^4+^, and Mo^5+^ by the removal of the surface passivated oxide layers through H_2_ treatment at 300 °C. The reduced low‐valence Mo species were then oxidized by the introduction of CO_2_ gas, and again reduced by H_2_ treatment at room temperature (Figure 5). From the Mo K‐edge XANES spectra (Figure S15e), the absorption edge position of hcp‐PdMo shifted slightly to higher energy after exposure to CO_2_, but retuned completely to its original position after H_2_ treatment, which is well consistent with the XPS results. The structure derived from octahedral Mo sites (MoO_2_ and MoO_3_ coordination environments) is not observed in XANES spectra of hcp‐PdMo, indicating that the segregation to MoO_x_ from PdMo does not occur even after pure CO_2_ treatment. This indicates that the change in the oxidation state of Mo in h‐PdMo is attributed to the high surface coverage of O* species derived from CO_2_ decomposition rather than the formation of MoO_x_. On the other hand, MoO_x_ co‐exists on the surface of Pd/Mo_2_N catalyst (Figures S16c and S16e), but it showed much lower activity and higher activation energy than hcp‐PdMo catalyst (Figure S9). Thus, the reaction mechanism for methanol synthesis over hcp‐PdMo catalyst is completely different from Pd‐MoO_x_ catalyst system, which also suggests that Pd‐MoO_x_ is not formed on PdMo surface. The C 1s, O 1s, and N 1s regions were also recorded to examine the dynamics of anionic species in hcp‐PdMo during the reaction. It was recently reported^[^ ^24^ ^]^ that the catalytic performance for the RWGS reaction is strongly dependent on the surface structure of MoN_x_ catalysts, which dynamically changes between MoO_x_ and MoC_x_ depending on the gas environment. The dissociation of CO_2_ into CO on MoN_x_ catalysts, followed by the Boudouard reaction,^[^ ^39^, ^40^ ^]^ results in the formation of graphitic carbon, which leads to surface reconstruction into MoC_x_. In contrast, the surface of the hcp‐PdMo catalysts exhibit redox reactions (Figure 4b,d,e) instead of carbonization (Figure 4c). The presence of anions served to stabilize the overall structure of the intermetallic catalysts, which were not as vulnerable to structural reconstruction as the MoO_x_ and MoN_x_ supports. The oxidation and reduction of Mo sites are specifically caused by two factors, that is, O* species derived from dissociated CO_2_ and hydrogen species produced at adjacent Pd sites. Considering the significantly superior activity of the hcp‐PdMo/Mo_2_N catalyst over those of Pd/MoO_2_ and Pd/Mo_2_N, this may be attributed to the poor propagation of hydrogen activated by Pd across the interface between the metal and the support,^[^ ^41^, ^42^ ^]^ which hinders any synergistic effect between the support and the loaded metal. Therefore, the low‐temperature redox behavior of Mo is likely due to the extremely high H_2_ dissociation ability of Pd, and the regularly ordered arrangement of the hcp‐PdMo catalyst between Pd and Mo facilitates the delivery and spillover of H* from Pd to Mo sites, which results in the reduction of Mo. Overall, these results indicate that CO_2_ can be activated by redox reaction with Mo under the promotion of Pd, even at low temperatures, which is consistent with the spectroscopic analysis and catalytic performance results.

XPS analysis was also performed on unloaded Mo_2_N and MoO_2_ catalysts to further investigate the role of Pd. Figures S16 and S17 show XPS measurements for the pre‐reduced Mo_2_N and MoO_2_ catalysts with subsequent treatments under CO_2_ and H_2_ at room temperature. A slight increase in the peak near 235 eV (oxidation of Mo) was observed for the Mo_2_N catalyst after CO_2_ treatment, whereas no significant change was observed for the MoO_2_ catalyst. This suggests that although there is a possibility of CO_2_ activation in Mo_2_N, it is not as effective at activating CO_2_ as the hcp‐PdMo catalysts. Moreover, the low activity of Mo_2_N may be due to the vulnerability to carbonization^[^ ^24^ ^]^ (Figure S16b) and irreversible oxidation of surface Mo_2_N (Figure S16c). For MoO_2_, the high oxidation state of Mo appears to have limited the ability to activate CO_2_ at low temperatures. Considering that Mo in the Mo_2_N catalyst was found to be slightly oxidized at room temperature, XPS measurements were conducted at increased CO_2_ gas treatment temperature (Figure S18). This temperature increase led to more pronounced oxidation of Mo, which indicates that CO_2_ can be partially activated in the absence of Pd. However, the presence of Pd is necessary to reduce the activation energy and facilitate the redox reaction of Mo at low temperatures.

### DFT Calculations

**Figure 6:**
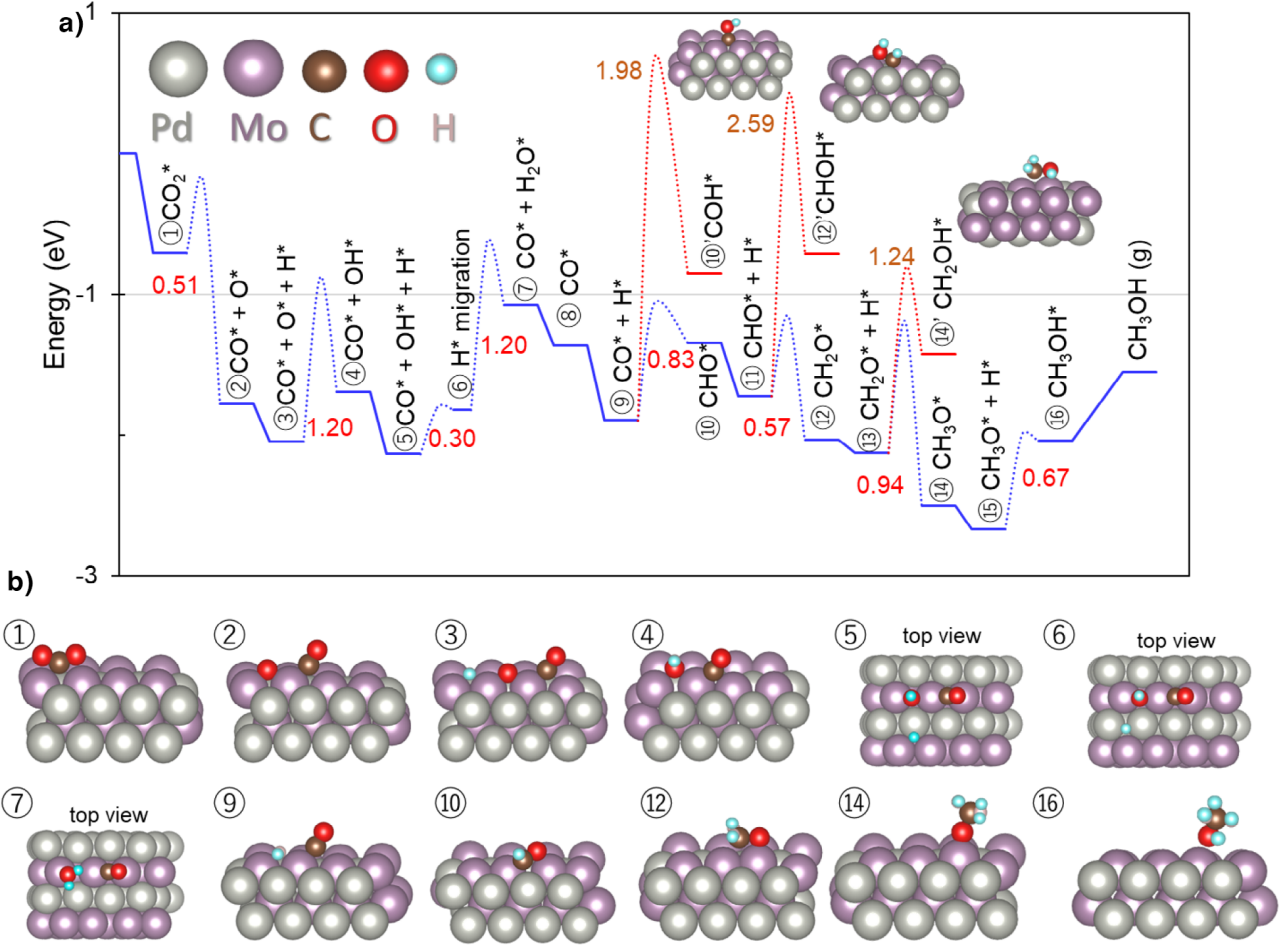
DFT calculations. a) Energy diagram for CO_2_ hydrogenation to CH_3_OH on PdMo(010) surface and the kinetic energy barrier of the key rate‐determining step. b) DFT‐optimized atomic structures for possible intermediates of CO_2_ hydrogenation to CH_3_OH over PdMo(010) model.

DFT calculations were performed to further clarify the reaction mechanism for CO_2_ hydrogenation over the hcp‐PdMo and Pd catalysts at the atomic level, where slab models for the PdMo(010) and Pd(111) surfaces were used for the calculations (Figure S19). First, possible stable surfaces for Pd and PdMo were investigated by calculating cleavage energy (Figure S20). The cleavage energy of PdMo(001) facet is lower than that of PdMo(010). However, as shown in Figure S21a, the adsorption energy of CO_2_ on the Pd‐terminated PdMo(001) surface (E_ads_ = −0.03 eV) is as low as that on pure Pd (E_ads_ = 0.07 eV), preventing further catalytic steps. On the other hand, the Mo‐terminated PdMo(001) surface suffers from strong oxophilicity, resulting in overly strong adsorption of intermediates (CO* and O*) and high energy barriers for OH* and H_2_O formation (Figure S21b). In contrast, the PdMo(010) surface features alternating Pd and Mo atoms, which alters the adsorption configuration of the intermediates and facilitates the hydrogenation of these intermediates, resulting in the significantly reduced energy barrier for each reaction step. It can thus be concluded that the PdMo(010) surface to serve as the active site for low‐temperature methanol synthesis. Furthermore, the PdMo(010) surface with the atomically interleaved structure of Mo and Pd was confirmed by HAADF‐STEM imaging (Figure S22). Figure S23 shows the results of comparative calculations conducted to investigate the activation of CO_2_, which involved the three main proposed reaction mechanisms, that is, the direct dissociation of CO_2_ to CO* and O*, the hydrogenation of O sites of CO_2_ to form carboxylate (COOH*), and the hydrogenation of C sites of CO_2_ to form HCOO* species. A H_2_ molecule is first dissociated and captured at a PdMoMo hollow site. CO_2_ is then adsorbed at the Mo bridge site with a bending configuration, which leads to its direct decomposition into CO* and O* with a lower activation energy of 0.51 eV. In contrast, the energy barriers for the formation of t‐COOH*, m‐HCOO*, and b‐HCOO* were calculated to be 1.31 , 1.34 , and 3.00 eV, respectively. The low activation energy barrier for the direct CO_2_ decomposition route indicates that CO_2_ is reduced through redox reactions with Mo, as determined by DRIFT experiments and X‐ray spectroscopy, which results in C ═ O bond breaking of CO_2_ to generate CO* and O*. The energy diagram for all elementary steps in methanol synthesis over the hcp‐PdMo catalyst is illustrated in Figure 6. Among all reaction steps, the energy barrier for water formation (step 6→7, *OH + H* → H_2_O*) is the highest (1.20 eV), making it the rate‐determining step of CO_2_ hydrogenation into methanol on the PdMo catalyst. Given the strong oxophilicity of Mo, it is reasonable to assume that breaking Mo‐O bond to form water and bare Mo site is a key step for the continuous catalytic cycle. Other reported Mo‐based catalysts also exhibit high energy barriers for both water formation and desorption.^[^ ^23^, ^24^, ^25^, ^26^ ^]^ For instance, the water formation energy is 1.81 eV for α‐Mo_2_C, while the desorption energy exceeds 2 eV for Pt–MoO_x_/Mo_2_N, which may lead to catalyst poisoning. While slight hydroxylation was observed in the IR experiment (Figure S24a) over the spent catalyst, the XANES spectra (Figure S24b) showed no significant changes in the oxidation states of Mo or Pd, indicating that the electronic state of the PdMo catalyst surface is preserved under the reaction condition. In contrast, the electronic state of Mo changes upon switching H_2_/CO_2_ (Figure S15b), indicating a dynamic redox response of the catalyst to reaction conditions. This reversible redox behavior is likely contributing the excellent stability and sustained low‐temperature activity of the hcp‐PdMo catalyst. After water desorption, we further investigated the subsequent hydrogenation of CO. Due to the complexity of the CO hydrogenation reaction, two different pathways in the three steps for the hydrogenation of intermediates were considered here, that is, the insertion of H* species to the C or O atom of CO* to form formyl (CHO*) and formaldehyde (CH_2_O*). More detailed DFT calculations revealed that the three steps for the hydrogenation of CO* were preferential on the C atom (Figure 6a, blue line) rather than the direct formation of hydroxyl (O─H) intermediates, such as the COH*, CHOH*, and CH_2_OH* species (Figure 6a, red line). The much lower activation barriers for the formation of HCO*, CH_2_O*, and CH_3_O* intermediates indicated that the H* species are more easily transferred from PdMoMo hollow sites to the corresponding intermediates. The reaction pathway from CO to CH_3_OH on Pd was shown in Figure S25. These results revealed that the rate‐determining step on Pd is the first hydrogenation step of CO* to CHO*, which has an activation energy that is approximately 0.65 eV higher than that on hcp‐PdMo. In the subsequent hydrogenation steps beyond formation of the CHO* intermediate, the activation energies on Pd are not significantly different from those on hcp‐PdMo. It can be inferred that the subsequent hydrogenation steps after the formation of the CHO* intermediate are relatively facile for both the Pd and hcp‐PdMo catalysts. Therefore, the hcp‐PdMo catalyst, which contains highly active CO* species, enables efficient hydrogenation at low temperatures, which effectively addresses the difficulty in the formation of the CHO* intermediate typically encountered with conventional Pd catalysts.

## Conclusion

The conditions for the synthesis of the hcp‐PdMo catalyst were optimized, and the location of N/O anions and manipulation of the mechanism by which the hcp‐PdMo catalyst exhibits excellent activity for methanol synthesis at low temperatures were investigated. XRD measurements indicated lattice expansion of hcp‐PdMo, which suggested the infiltration of anions into the lattice, a finding that was corroborated by XPS depth profile results. Furthermore, the EXAFS and TPD results confirmed that N^3−^ ions were predominantly present around Mo, which contributes to the stabilization of the hcp‐PdMo intermetallic compound structure. Compared to the random solid solution alloy of the fcc‐PdMo catalyst, the regularly ordered arrangement of the hcp‐PdMo catalyst contributes to enhanced selectivity for the CO_2_ hydrogenation reaction at low reaction temperatures. The (101) plane of regularly stacked Pd and Mo atoms is the crucial active surface for methanol production. The catalytic performance could be significantly improved by an increase of the surface area of the hcp‐PdMo catalysts. DRIFTS measurements suggested that the reaction mechanism of the hcp‐PdMo catalyst may follows the R&C mechanism, where CO_2_ is activated at Mo sites, and CO* species adsorbed on Mo are further hydrogenated to form CH_3_O*. Structural analysis via XPS and XAFS revealed that the ordered structure of the hcp‐PdMo catalyst enhances the transfer and spillover of H* from Pd to Mo sites, which facilitates the reduction of Mo at room temperature and accounts for the continuous production of methanol. This study not only presents a novel approach for the synthesis of metastable intermetallic compounds with outstanding low‐temperature methanol synthesis performance, but also paves the way for innovative catalyst design aimed at the efficient utilization of CO_2_ as a resource.

## Supporting Information

The authors have cited additional references within the Supporting Information.

## Conflict of Interests

The authors declare no conflict of interest.

## Supporting information



Supplementary Information

## Data Availability

The data that support the findings of this study are available from the corresponding author upon reasonable request.

## References

[1] M. Aresta , A. Dibenedetto , A. Angelini , Chem. Rev. 2014, 114, 1709–1742. 24313306 10.1021/cr4002758

[2] S. Navarro‐Jaén , M. Virginie , J. Bonin , M. Robert , R. Wojcieszak , A. Y. Khodakov , Nat. Rev. Chem. 2021, 5, 564–579. 37117584 10.1038/s41570-021-00289-y

[3] A. Álvarez , A. Bansode , A. Urakawa , A. V. Bavykina , T. A. Wezendonk , M. Makkee , J. Gascon , F. Kapteijn , Chem. Rev. 2017, 117, 9804–9838. 28656757 10.1021/acs.chemrev.6b00816 PMC5532695

[4] S. T. Bai , G. De Smet , Y. Liao , R. Sun , C. Zhou , M. Beller , B. U. W. Maes , B. F. Sels , Chem. Soc. Rev. 2021, 50, 4259–4298. 33687387 10.1039/d0cs01331e

[5] S. Kattel , P. J. Ramírez , J. G. Chen , J. A. Rodriguez , P. Liu , Science (1979) 2017, 355, 24. 10.1126/science.aal3573 28336665

[6] Y . Xu , Z. Gao , Y. Xu , X. Qin , X. Tang , Z. Xie , J. Zhang , C. Song , S. Yao , W. Zhou , D. Ma , L. Lin , Appl. Catal. B 2024, 344, 123656.

[7] C. Huang , S. Zhang , W. Wang , H. Zhou , Z. Shao , L. Xia , H. Wang , Y. Sun , ACS Catal. 2024, 14, 1324–1335.

[8] M. Zabilskiy , V. L. Sushkevich , D. Palagin , M. A. Newton , F. Krumeich , J. A. van Bokhoven , Nat. Commun. 2020, 11, 2409. 32415106 10.1038/s41467-020-16342-1 PMC7229192

[9] H. Sugiyama , N. Nakamura , S. Watanabe , J. Kim , M. Kitano , H. Hosono , J. Phys. Chem. Lett. 2023, 14, 1259–1264. 36719321 10.1021/acs.jpclett.2c03427

[10] H. Kong , H. Y. Li , G. D. Lin , H. B. Zhang , Catal Letters 2011, 141, 886–894.

[11] A. Ota , E. L. Kunkes , I. Kasatkin , E. Groppo , D. Ferri , B. Poceiro , R. M. Navarro Yerga , M. Behrens , J. Catal. 2012, 293, 27–38.

[12] F. Studt , I. Sharafutdinov , F. Abild‐Pedersen , C. F. Elkjær , J. S. Hummelshøj , S. Dahl , I. Chorkendorff , J. K. Nørskov , Nat. Chem. 2014, 6, 320–324. 24651199 10.1038/nchem.1873

[13] S. R. Docherty , C. Copéret , J. Am. Chem. Soc. 2021, 143, 6767–6780. 33942617 10.1021/jacs.1c02555

[14] O. Martin , A. J. Martín , C. Mondelli , S. Mitchell , T. F. Segawa , R. Hauert , C. Drouilly , D. Curulla‐Ferré , J. Pérez‐Ramírez , Angew. Chem. Int. Ed. 2016, 128, 6369–6373. 10.1002/anie.201600943 26991730

[15] J. Wang , G. Li , Z. Li , C. Tang , Z. Feng , H. An , H. Liu , T. Liu , C. Li , Sci. Adv. 2017, 3, e1701290. 28989964 10.1126/sciadv.1701290 PMC5630239

[16] M. Wang , L. Zheng , G. Wang , J. Cui , G.‐L. Guan , Y.‐T. Miao , J.‐F. Wu , P. Gao , F. Yang , Y. Ling , X. Luo , Q. Zhang , G. Fu , K. Cheng , Y. Wang , J. Am. Chem. Soc. 2024, 146, 14528–14538. 38742912 10.1021/jacs.4c00981

[17] A. J. Poerjoto , J. Ashok , N. Dewangan , S. Kawi , J. CO2 Util. 2021, 47, 101498.

[18] R. Kanega , N. Onishi , S. Tanaka , H. Kishimoto , Y. Himeda , J. Am. Chem. Soc. 2021, 143, 1570–1576. 33439639 10.1021/jacs.0c11927

[19] J. Hu , L. Yu , J. Deng , Y. Wang , K. Cheng , C. Ma , Q. Zhang , W. Wen , S. Yu , Y. Pan , J. Yang , H. Ma , F. Qi , Y. Wang , Y. Zheng , M. Chen , R. Huang , S. Zhang , Z. Zhao , J. Mao , X. Meng , Q. Ji , G. Hou , X. Han , X. Bao , Y. Wang , D. Deng , Nat. Catal. 2021, 4, 242–250.

[20] H. Sugiyama , M. Miyazaki , M. Sasase , M. Kitano , H. Hosono , J. Am. Chem. Soc. 2023, 145, 9410–9416. 36995761 10.1021/jacs.2c13801 PMC10161205

[21] M. S. Duyar , C. Tsai , J. L. Snider , J. A. Singh , A. Gallo , J. S. Yoo , A. J. Medford , F. Abild‐Pedersen , F. Studt , J. Kibsgaard , S. F. Bent , J. K. Nørskov , T. F. Jaramillo , Angew. Chem. Int. Ed. 2018, 130, 15265–15270. 10.1002/anie.201806583 30134041

[22] H. Li , L. Wang , Y. Dai , Z. Pu , Z. Lao , Y. Chen , M. Wang , X. Zheng , J. Zhu , W. Zhang , R. Si , C. Ma , J. Zeng , Nat. Nanotechnol. 2018, 13, 411–417. 29556007 10.1038/s41565-018-0089-z

[23] H. X. Liu , J. Y. Li , X. Qin , C. Ma , W. W. Wang , K. Xu , H. Yan , D. Xiao , C. J. Jia , Q. Fu , D. Ma , Nat. Commun. 2022, 13, 5800. 36192383 10.1038/s41467-022-33308-7 PMC9530113

[24] H. Xin , R. Li , L. Lin , R. Mu , M. Li , D. Li , Q. Fu , X. Bao , Nat. Commun. 2024, 15, 3100. 38600159 10.1038/s41467-024-47550-8 PMC11271606

[25] M. Ahmadi Khoshooei , X. Wang , G. Vitale , F. Formalik , K. O. Kirlikovali , R. Q. Snurr , P. Pereira‐Almao , O. K. Farha , Science 2024, 384, 540–546. 38696554 10.1126/science.adl1260

[26] G. Liu , P. Liu , D. Meng , T. Zhao , X. Qian , Q. He , X. Guo , J. Qi , L. Peng , N. Xue , Y. Zhu , J. Ma , Q. Wang , X. Liu , L. Chen , W. Ding , Nat. Commun. 2023, 14, 513. 36720869 10.1038/s41467-023-36259-9 PMC9889347

[27] A. El‐Himri , F. Sapiña , R. Ibañez , A. Beltrán , J. Mater. Chem. 2001, 11, 2312–2315.

[28] M. S. Frei , C. Mondelli , R. García‐Muelas , K. S. Kley , B. Puértolas , N. López , O. V. Safonova , J. A. Stewart , D. Curulla Ferré , J. Pérez‐Ramírez , Nat. Commun. 2019, 10, 3377. 31358766 10.1038/s41467-019-11349-9 PMC6662860

[29] K. Lee , U. Anjum , T. P. Araújo , C. Mondelli , Q. He , S. Furukawa , J. Pérez‐Ramírez , S. M. Kozlov , N. Yan , Appl. Catal. B 2022, 304, 120994.

[30] N. Lawes , I. E. Gow , L. R. Smith , K. J. Aggett , J. S. Hayward , L. Kabalan , A. J. Logsdail , T. J. A. Slater , M. Dearg , D. J. Morgan , N. F. Dummer , S. H. Taylor , M. Bowker , C. R. A. Catlow , G. J. Hutchings , Faraday Discuss. 2022, 242, 193–211. 10.1039/d2fd00119e 36189732

[31] J. A. Rodriguez , J. Phys. Chem. 1994, 98, 5758–5764.

[32] G. Wowsnick , D. Teschner , I. Kasatkin , F. Girgsdies , M. Armbrüster , A. Zhang , Y. Grin , R. Schlögl , M. Behrens , J. Catal. 2014, 309, 209–220.

[33] J. Liu , J. L. Duan , M. E. Toimil‐Molares , S. Karim , T. W. Cornelius , D. Dobrev , H. J. Yao , Y. M. Sun , M. D. Hou , D. Mo , Z. G. Wang , R. Neumann , Nanotechnology 2006, 17, 1922–1926.

[34] F. C. Meunier , C. Scarfiello , Appl. Catal. B 2023, 330, 122610.

[35] L. S. Rothman , L. D. G. Young , J. Quonf. Speclrosc. Radial. Transfer 1981, 25, 505–524.

[36] T. P. Araújo , J. Morales‐Vidal , G. Giannakakis , C. Mondelli , H. Eliasson , R. Erni , J. A. Stewart , S. Mitchell , N. López , J. Pérez‐Ramírez , Angew. Chem. Int. Ed. 2023, 62, e202306563. 10.1002/anie.202306563 37395462

[37] Z. Cai , J. Dai , W. Li , K. B. Tan , Z. Huang , G. Zhan , J. Huang , Q. Li , ACS Catal. 2020, 10, 13275–13289.

[38] T. Kecskés , R. Németh , J. Raskó , J. Kiss , Vacuum 2005, 80, 64–68.

[39] T. Osaki , T. Mori , React. Kinet. Catal. Lett. 2006, 89, 333–339.

[40] F. Xing , Y. Nakaya , S. Yasumura , K. Shimizu , S. Furukawa , Nat. Catal. 2022, 5, 55–65.

[41] R. Prins , Chem. Rev. 2012, 112, 2714–2738. 22324402 10.1021/cr200346z

[42] K. Lee , P. C. D. Mendes , H. Jeon , Y. Song , M. P. Dickieson , U. Anjum , L. Chen , T.‐C. Yang , C.‐M. Yang , M. Choi , S. M. Kozlov , N. Yan , Nat. Commun. 2023, 14, 819. 36781851 10.1038/s41467-023-36407-1 PMC9925737

