# Supplementary material for: Origin of Room Temperature Methanol Synthesis over Hcp‐PdMo
Source: Angew Chem Int Ed Engl. 2025 Jun 3;64(32):e202505634. doi: 10.1002/anie.202505634 (PMC12322628; doi:10.1002/anie.202505634)
Supplement: Supplementary file 1 — Supplementary Information [file ANIE-64-e202505634-s001.docx]

Supporting Information

Origin of Room Temperature Methanol Synthesis over Hcp-PdMo

Feilong Xing^†^, Ruopeng Wang^†^, Shiyao Wang^†^, Hironobu Sugiyama^†^, Chenyang Zhu^†^, Masayoshi Miyazaki^†^, Hideo Hosono^†, §,^ *, and Masaaki Kitano^†, ‡,^ *

^†^ *MDX Research Center for Element Strategy, Institute of Integrated Research, Institute of Science Tokyo, 4259 Nagatsuta, Midori-ku, Yokohama 226-8503, Japan*

^‡^ *Advanced Institute for Materials Research (WPI-AIMR), Tohoku University, Sendai 980-8577, Japan*

^§^ *International Center for Materials Nanoarchitectonics (WPI-MANA), National Institute for Materials Science (NIMS), Tsukuba, Ibaraki 305-0044, Japan*

**Corresponding authors**

Email: hosono.h.aa@m.titech.ac.jp

Email: [kitano.m.aa@m.titech.ac.jp](mailto:kitano.m.aa@m.titech.ac.jp)

**Experimental section**

**Catalysts preparation**

hcp-PdMo, hcp-PdMo/Mo_2_N, and Mo_2_N catalysts were prepared via ammonolysis of an oxide precursor. Oxide precursors were prepared by the Pechini method using (NH_4_)_6_Mo_7_O_24_·4H_2_O (81-83% MoO_3_ basis, Aldrich) and Pd(CH_3_COO)_2_ (98%, TCI) as Mo and Pd sources, respectively. Mo and Pd sources in predetermined ratios and citric acid at twice the amount of the total metal ions were dissolved in 6% aqueous HNO_3_ at room temperature. The solution mixture was placed in a heating mantle at 80 °C, stirred, and evaporated until a transparent gel formed. The temperature was then raised to 200 °C and the gel was converted to an amorphous precursor, which was calcined at 500 °C for 2 h in air to obtain the oxide precursor. Finally, ammonolysis of the oxide precursor was performed at 500~950 °C in a flow of NH_3_ (10 mL min^−1^) for 12 h. Same method as above, the high surface area (HSA) hcp-PdMo catalysts prepared by using polyvinylpyrrolidone (K30, Fujifilm Wako) as a chelating agent instead of citric acid. The (002) preferred orientation hcp-PdMo catalyst was prepared by a process of ammonolysis of a physical mixture of palladium precursor and MoO_3_ at 700°C. The resultant material with 55wt% Pd is referred to as hcp-PdMo, whereas that with 5wt% Pd is named as 5wt% hcp-PdMo/Mo_2_N because hcp-PdMo intermetallic nanoparticles are formed on Mo_2_N. Mo_2_N was prepared from Mo-oxide without Pd by the same procedure as hcp-PdMo. For 5wt% Pd/Mo_2_N and 5wt% Pd/MoO_2_ catalyst, Pd(acac)_2_ (99%, Aldrich) was used as a Pd precursor, which was mixed with Mo_2_N or MoO_2_ in an agate mortar. The mixture was then heated at 300 °C in a flow of H_2_ (10 mL min^−1^) for 2 h with a heating rate of 2 °C min^−1^. The copper-based methanol synthesis catalyst (Cu/ZnO/Al_2_O_3_ pellets, Alfa Aesar) was obtained as a commercially available product. Cu/ZnO/ Al_2_O_3_ pellets were hand-milled in an agate mortar and used as a powder. All catalysts were reduced by H_2_ at 300 °C for 2 h with a heating rate of 2 °C min^−1^ before the CO_2_ hydrogenation reaction.

**Characterization**

XRD (D2 PHASER, Bruker) with Cu Kα radiation (λ = 0.15418 nm) was used to confirm the crystal structure and purity. The sample was placed in an air-isolated transparent capsule for measurements. The microstructural characteristics of the samples were determined using transmission electron microscopy (TEM; JEM-ARM200F, Jeol). EDX mapping of the same area was also performed. Pd K and Mo K-edge X-ray adsorption fine structure (XAFS) data were obtained using the synchrotron radiation ring at the PF-12C and NW-10A beamlines of the KEK Photon Factory with Si(111) single-crystal monochromators. Athena and Artemis softwares v.0.9.25 implemented in the Demeter package were used for the analysis of the obtained XAFS spectra. Before the measurement, the hcp-PdMo single-phase catalyst was pretreated with H_2_ (10 mL min^−1^) at 300 °C for 2 h in a quartz tube, followed by cooling to room temperature with Ar purge (10 ml min^−1^) and stored in the glovebox. Part of the sample was stored in a glove box, while the rest was exposed to CO_2_ at room temperature, and another portion was exposed to CO_2_+H_2_ at room temperature. Additionally, part of the CO_2_ exposed sample was further treated with H_2_ at room temperature. XPS (Kratos-ULTRA2, Shimadzu) analysis was conducted using an air-isolated sample vessel and an apparatus equipped with a charge neutralization system. The binding energy was corrected against the signal of C 1s orbital (C 1s = 284.6 eV). Before the measurement, the air-exposed hcp-PdMo single-phase catalyst was pretreated with H_2_ (10 mL min^−1^) at 300 °C for 2 h in a quartz tube, followed by cooling to room temperature with Ar purge (10 ml min^−1^) and stored in the glovebox. Part of the sample was stored in a glove box, while the rest was exposed to CO_2_ at room temperature. Then, part of the CO_2_ exposed sample was further treated with H_2_ at room temperature. TPD experiments (BELSORP-miniII, BEL) were conducted at a heating rate of 10°C min^−1^ in a stream of Ar gas (30 mL min^−1^), and the species released from the sample were monitored with a mass spectrometer (BELMass, MicrotracBEL, Japan). For CO_2_-TPSR experiment, the h-PdMo catalyst was pretreated under H_2_ at 300 °C for 2 hours, then cooled to room temperature under He for 1 hour to stabilize the signal. The MS signal was recorded after introducing 5% CO_2_/He. The Brunauer-Emmett-Teller (BET) specific surface areas of the support materials were obtained from nitrogen adsorption-desorption isotherms measured at 77K using an automatic gas adsorption instrument (BELSORP-mini II, MiccrotracBEL). Diffuse reflectance infrared Fourier transform (DRIFT) spectroscopy was performed using a spectrometer (FT/IR-6X, JASCO) with a mercury-cadmium-tellurium (MCT) detector at a resolution of 4 cm^−1^. Prior to CO_2_ hydrogenation, the 5wt% hcp-PdMo/Mo_2_N catalysts were reduced by H_2_ at 300 °C for 2 h. After the sample was cooled to room temperature, a mixed gas (CO_2_:H_2_ = 1:3, 20 mL min^−1^) or CO_2_/H_2_ switched gas was supplied to the chamber, and measurements were conducted at room temperature. The HCOOH adsorption reaction conditions were as follows: the sample was vacuumed after H_2_ pretreatment, formic acid was introduced, and the vacuum was pulled again to confirm the adsorption of formic acid. Then, 15 mL min^-1^ H_2_ was purged for 45 min at room temperature.

**Catalytic test**

Methanol synthesis activity was evaluated in a quartz/steel fixed-bed reactor. The catalyst (100 mg) was weighed and packed in the tube, treated with a flow rate of H_2_ was set to 30 mL min^-1^ at 300 °C for 2 h. The reaction was then carried out by feeding reactant gas mixture (CO_2_: H_2_: Ar = 1:3:1, a total flow rate of 50 mL min^-1^), and WHSV = 30000 mL g_cat_^-1^ h^-1^. The reaction was conducted under both atmospheric and pressurized conditions, with the reaction temperature ramped up from 100 °C to 200 °C at a rate of 2 °C min^-1^, and measurements were taken at 20 °C intervals. The gas phase was analyzed and quantified using an online gas chromatograph (Agilent 7890A) equipped with a thermal conductivity detector (TCD, column: SHINCARBON ST) and a flame ionization detector (FID, column: DB-1(30 m×320 μm×3 μm), and Hayasep Q). For all the catalysts, CH_3_OH, CH_4_, CO, CO_2_, H_2_O and H_2_ were detected as reaction products in outlet gas. Due to the difficulty in quantifying tiny changes in CO_2_ flow rate, especially those below 1%, the CO_2_ conversion was calculated based on the total amount of generated products when the conversion was below 1%.

**DFT calculation**

Density functional theory (DFT) calculations were conducted using the Revised-Perdew-Burke-Ernzerhof (RPBE) functional implemented in the Vienna Ab initio Simulation Package (VASP) ^1,2^. The cutoff energy of the plane-wave basis function system is 550 eV, and the Monkhorst-Pack k-point grid for Brillouin zone sampling is 4 x 3 x 1. The z-axis was taken to be perpendicular to the surface. Energy and force convergence were based on 1.0 x 10^-5^ eV and 1.0 x 10^-2^ eV/Å, respectively. The Climbing image nudged elastic band (CI-NEB) method was used to calculate the activation barrier. VASPKIT^3^ and VESTA^4^ were used to create input files and visualize output files, respectively. 3×3×1 gamma-centered k-point meshes for (111) plane of the slab model of Pd and MoN. A supercell of (010) plane of PdMo with five layers was used, and we kept its last two layers frozen for surface calculation. A supercell with a 25 Å vacuum layer along the c-axis was constructed in the slab model to prevent the interaction between the periodically repeated slabs. Surface formation energies were calculated following eqs :

𝐸 = (𝐸_𝑠𝑙𝑎𝑏_ ― 𝐸_𝑏𝑢𝑙𝑘_)/2𝑆

where 𝐸_𝑏𝑢𝑙𝑘_ is the total energy of a unit cell of bulk model after structural optimization, 𝐸_𝑠𝑙𝑎𝑏_ is the total energy of a slab model, and S is the surface area of the exposed plane.

**Supporting Figures**

Figure S1. SEM-EDX image of Pd-Mo catalyst prepared at 600 °C.


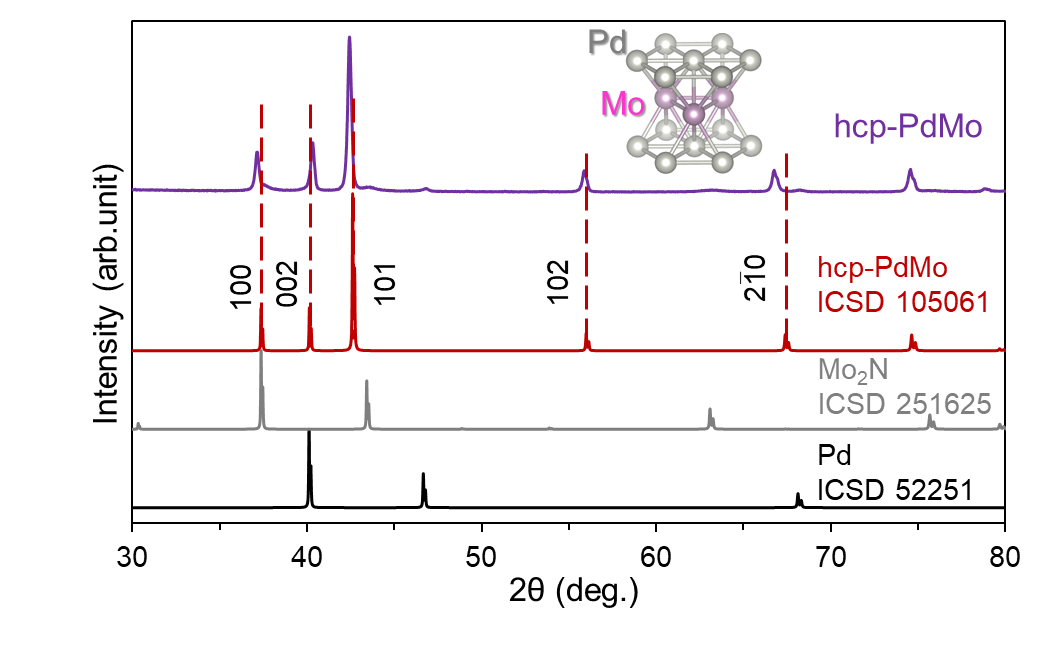


Figure S2. XRD pattern and structure of hcp-PdMo catalyst prepared at 700°C and reference data.


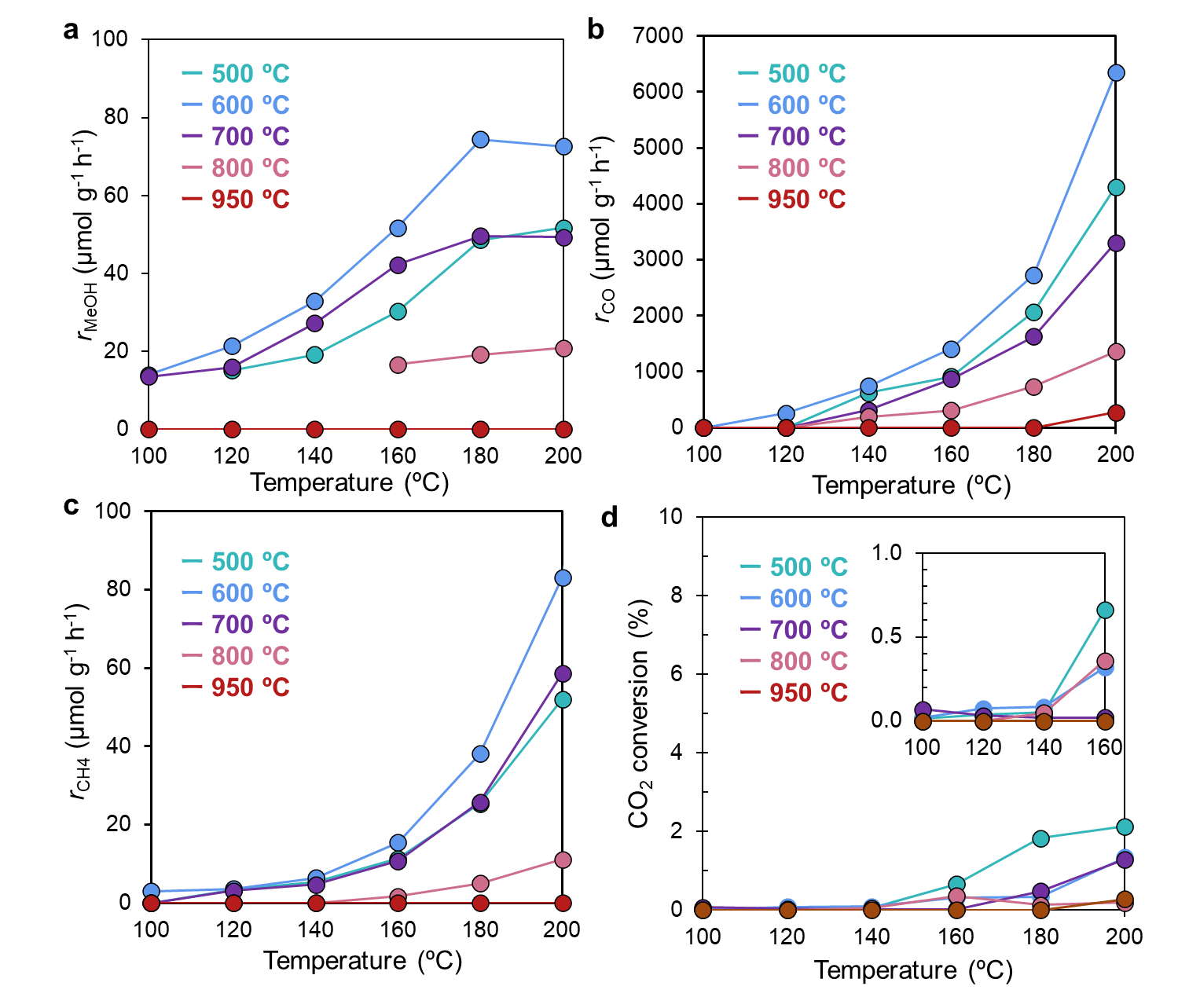


Figure S3 (a) MeOH, (b) CO, (c) CH_4_ production rate and (d) CO_2_ conversion over different temperature treatment PdMo catalysts. (Reaction conditions: 0.1 g catalyst, CO_2_: H_2_: Ar =10:30:10 mL min^−1^, 100-200 °C, 0.1 MPa)


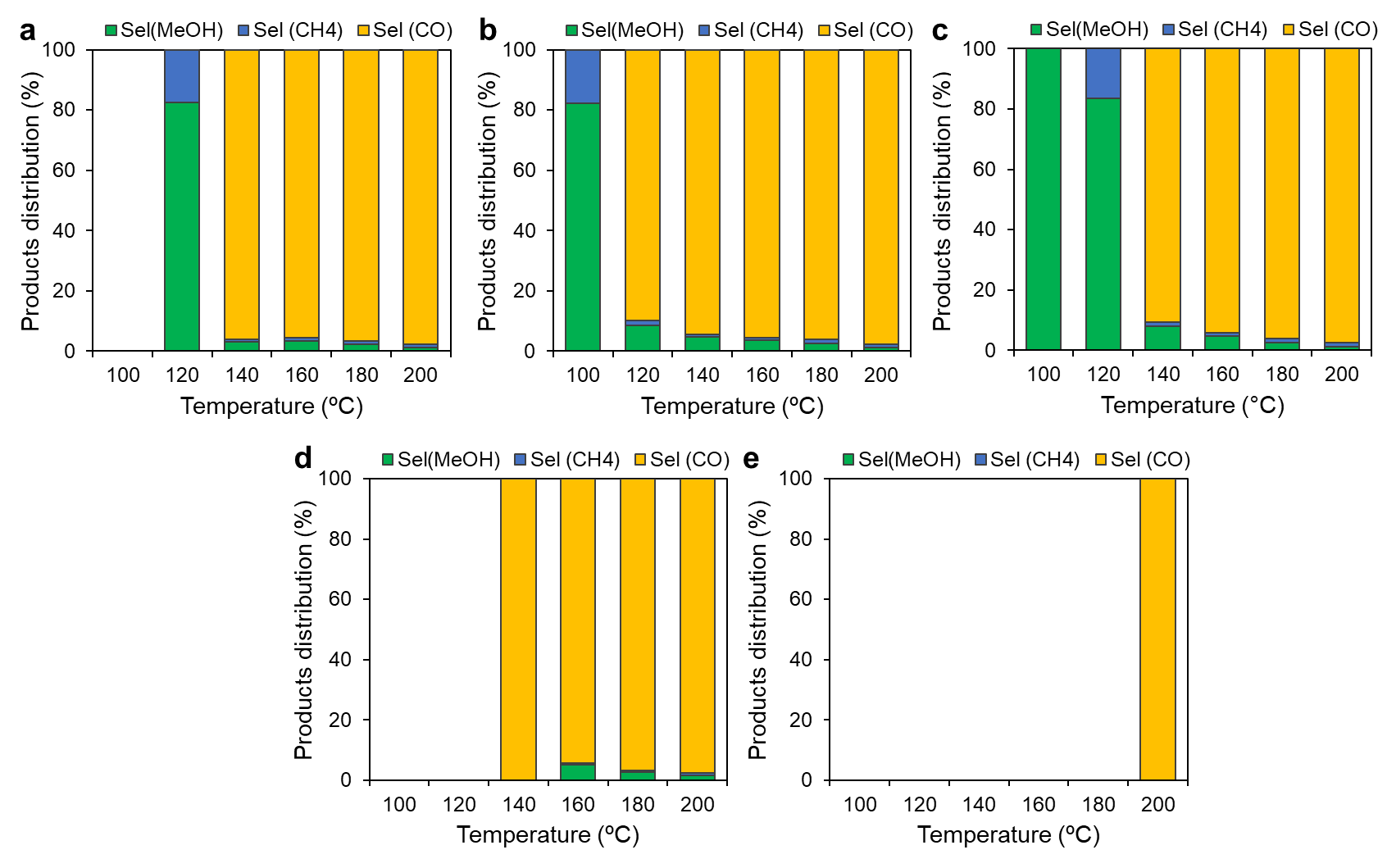


Figure S4 Selectivity distribution of PdMo catalysts prepared at different temperatures: (a)500°C, (b) 600°C, (c) 700°C, (d) 800°C, and (e) 950°C. (Reaction conditions: 0.1 g catalyst, CO_2_: H_2_: Ar =10:30:10 mL min^−1^, 100-200 °C, 0.1 MPa)

**
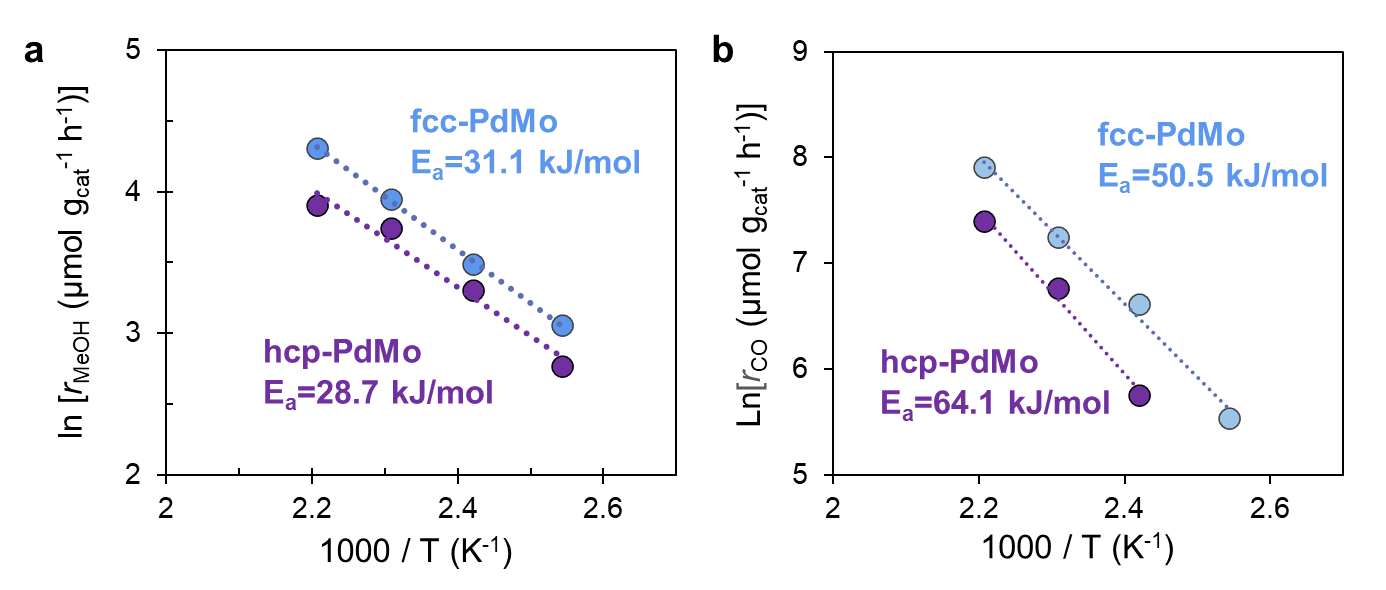
**

Figure S5. (a) Arrhenius plots of CH_3_OH and CO synthesis rate over the fcc-PdMo and hcp-PdMo catalysts


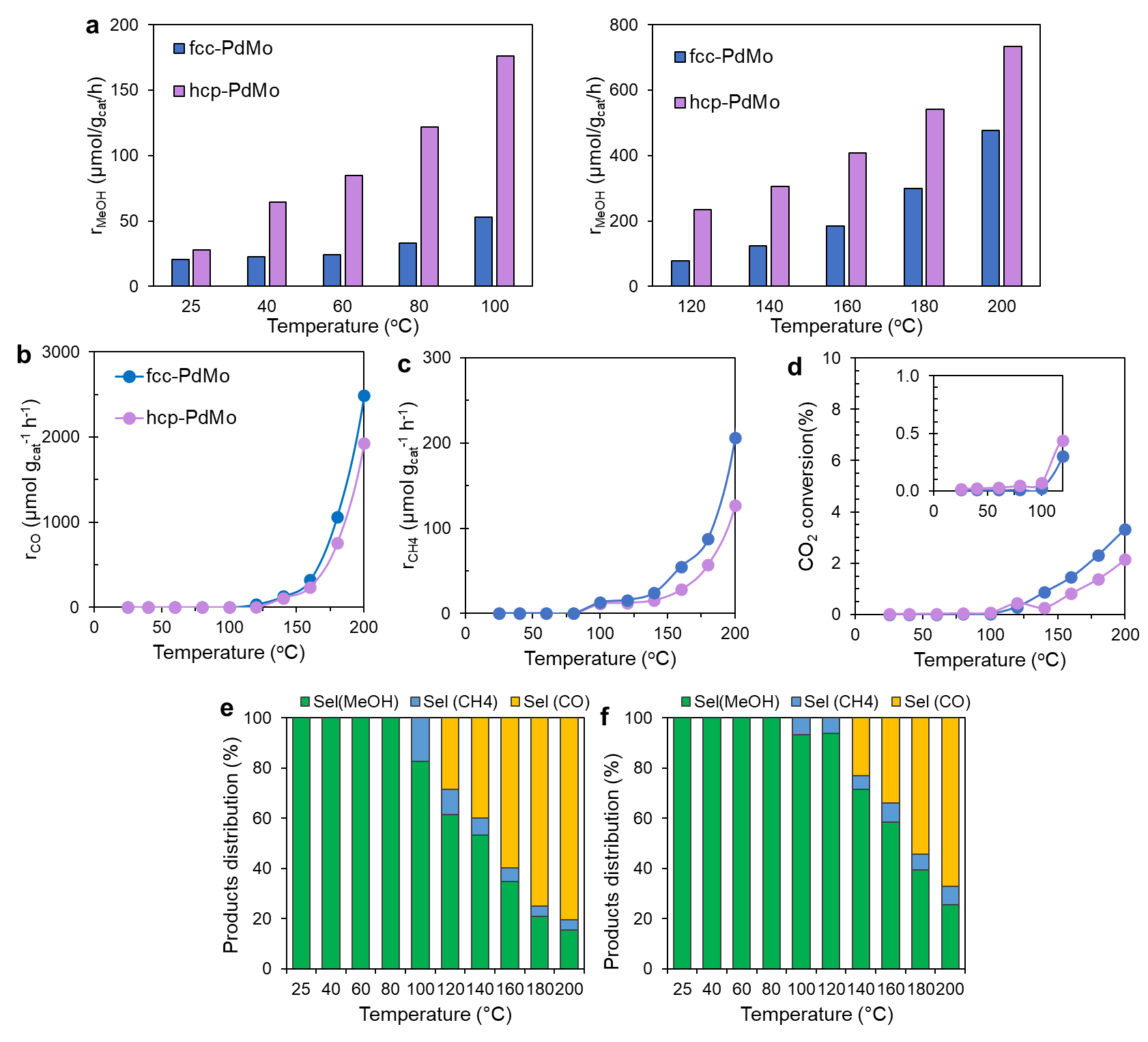


Figure S6 (a) MeOH, (b) CO, (c) CH_4_ production rate and (d) CO_2_ conversion over the fcc-PdMo and hcp-PdMo catalysts. Selectivity distribution of (e) fcc-PdMo and (f) hcp-PdMo (Reaction conditions: 0.1 g catalyst, CO_2_: H_2_: Ar =10:30:10 mL min^−1^, 25-200 °C, 0.9 MPa)

**
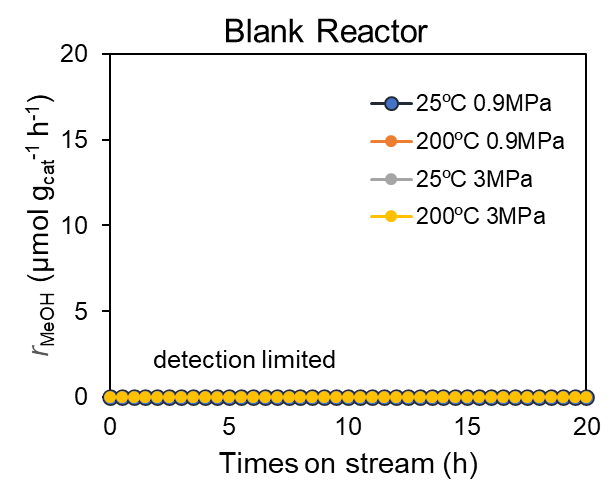
**

Figure S7 MeOH formation rate of the blank reactor under virous conditions.


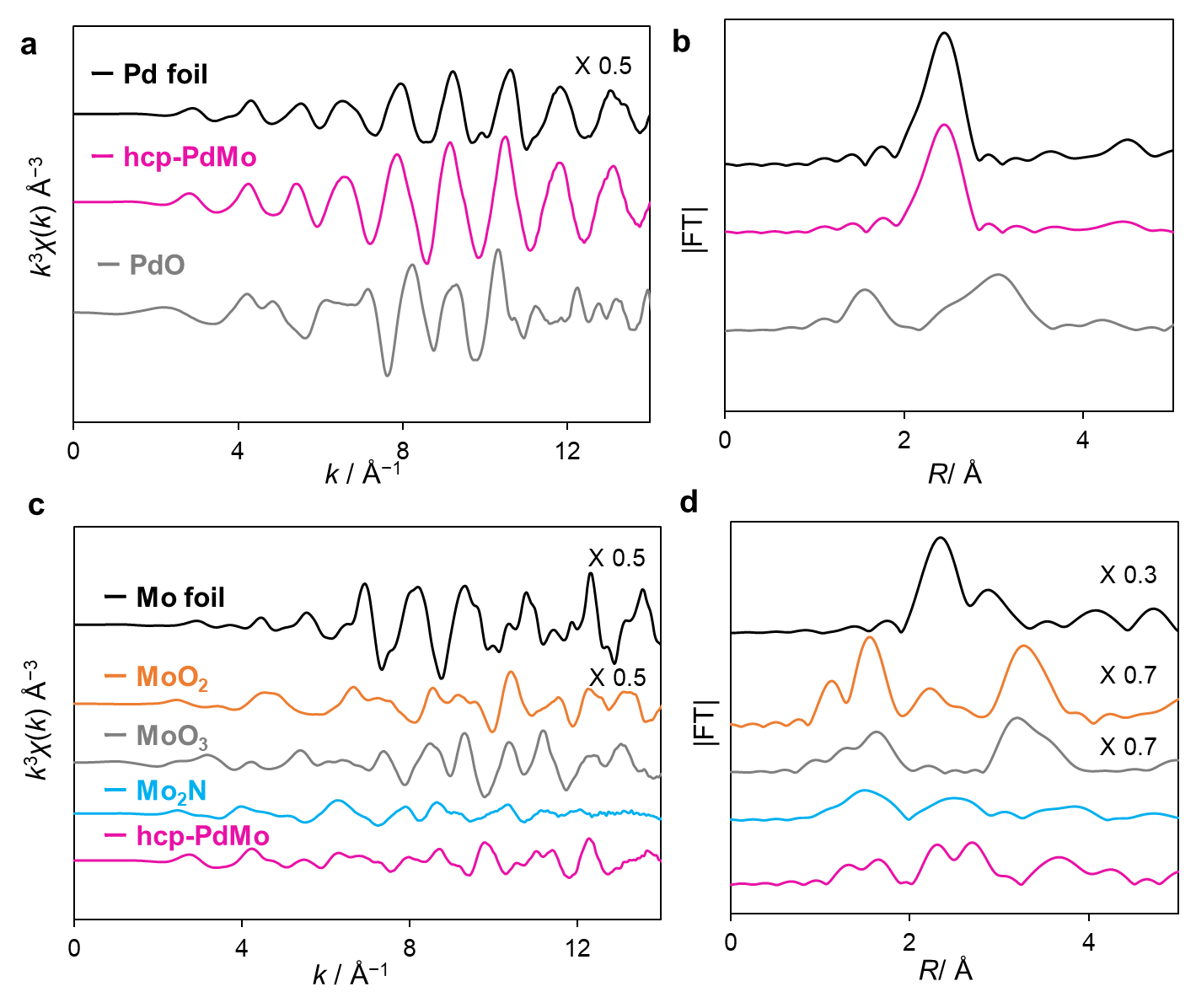


Figure S8. Extended X-ray absorption near edge structure (EXAFS) spectra of the hcp-PdMo catalysts and reference compounds, (a) Pd K-edge, (c) Mo K-edge. Fourier transform of EXAFS of the educed catalysts and reference compounds, (b) Pd K-edge, (d) Mo K-edge.

**
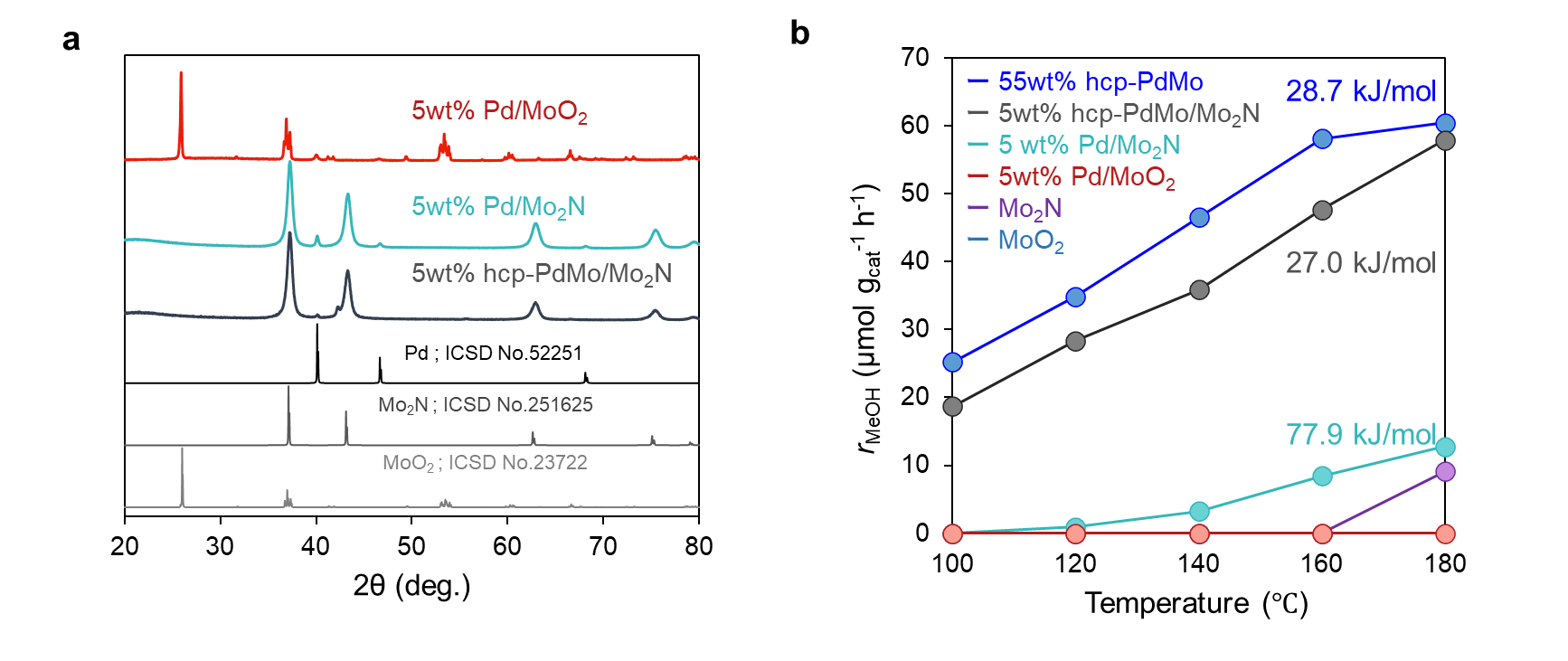
**

Figure S9 (a) XRD patterns of 5wt% hcp-PdMo/Mo_2_N and Pd-based catalysts (b) MeOH synthesis activity and apparent activation energy of 5wt% hcp-PdMo/Mo_2_N and Pd-based catalysts under atmospheric pressure.


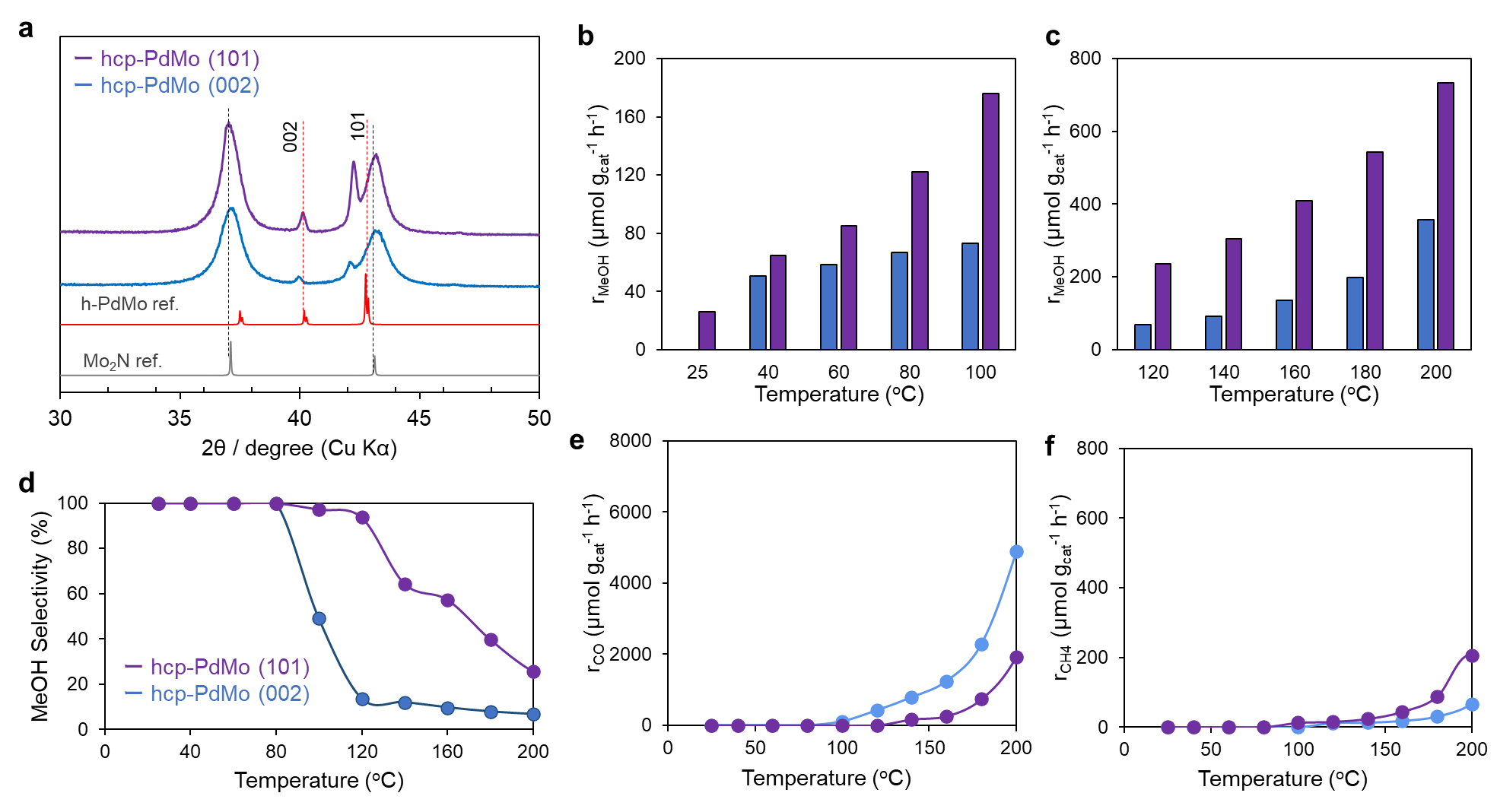


Figure S10 (a) XRD patterns of (101) preferred orientation catalysts (denoted as: hcp-PdMo(101)) and (002) preferred orientation catalysts (denoted as: hcp-PdMo(002)). Synthesis rate and selectivity of products as a function of reaction temperature at 0.9 MPa during CO_2_ hydrogenation over different orientation catalysts. (b)MeOH production rate in the range of 25-100°C, and (c) 100-200°C, (d) MeOH selectivity, (e) CO production rate, and (f) CH_4_ production rate.

The textural coefficients (TC)^19^ of reflections were calculated based on the intensities of (002) and (101) planes from XRD patterns for these two catalysts. The results are below:

**TC (101):** 0.9_hcp-PdMo(101)_ > 0.7_hcp-PdMo(002)_ and **TC (002):** 1.1_hcp-PdMo(101)_ < 1.3_hcp-PdMo(002)_.

The TC value for any crystal plane is 1 in an ideal polycrystalline sample, with higher TC values indicating more favorable crystal plane orientation.^20^

**
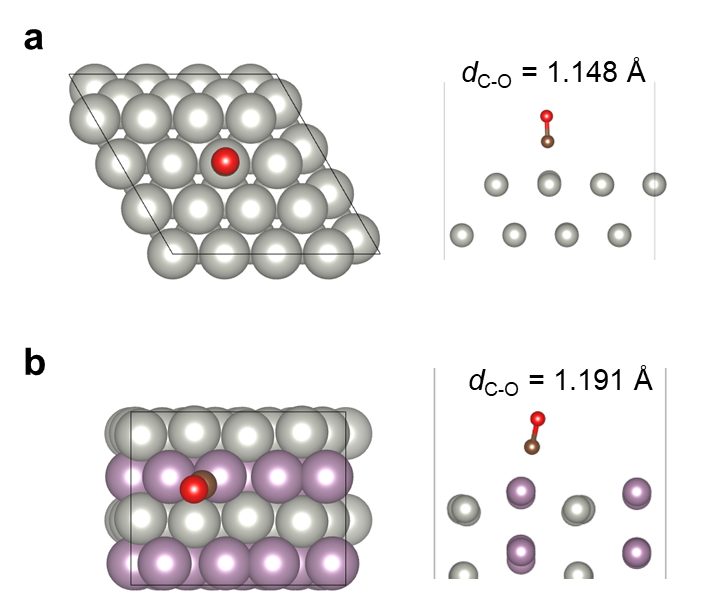
**

Figure S11 The structure and bond length of *CO adsorbed on the (a) Pd (111) and (b) PdMo (010) surfaces.


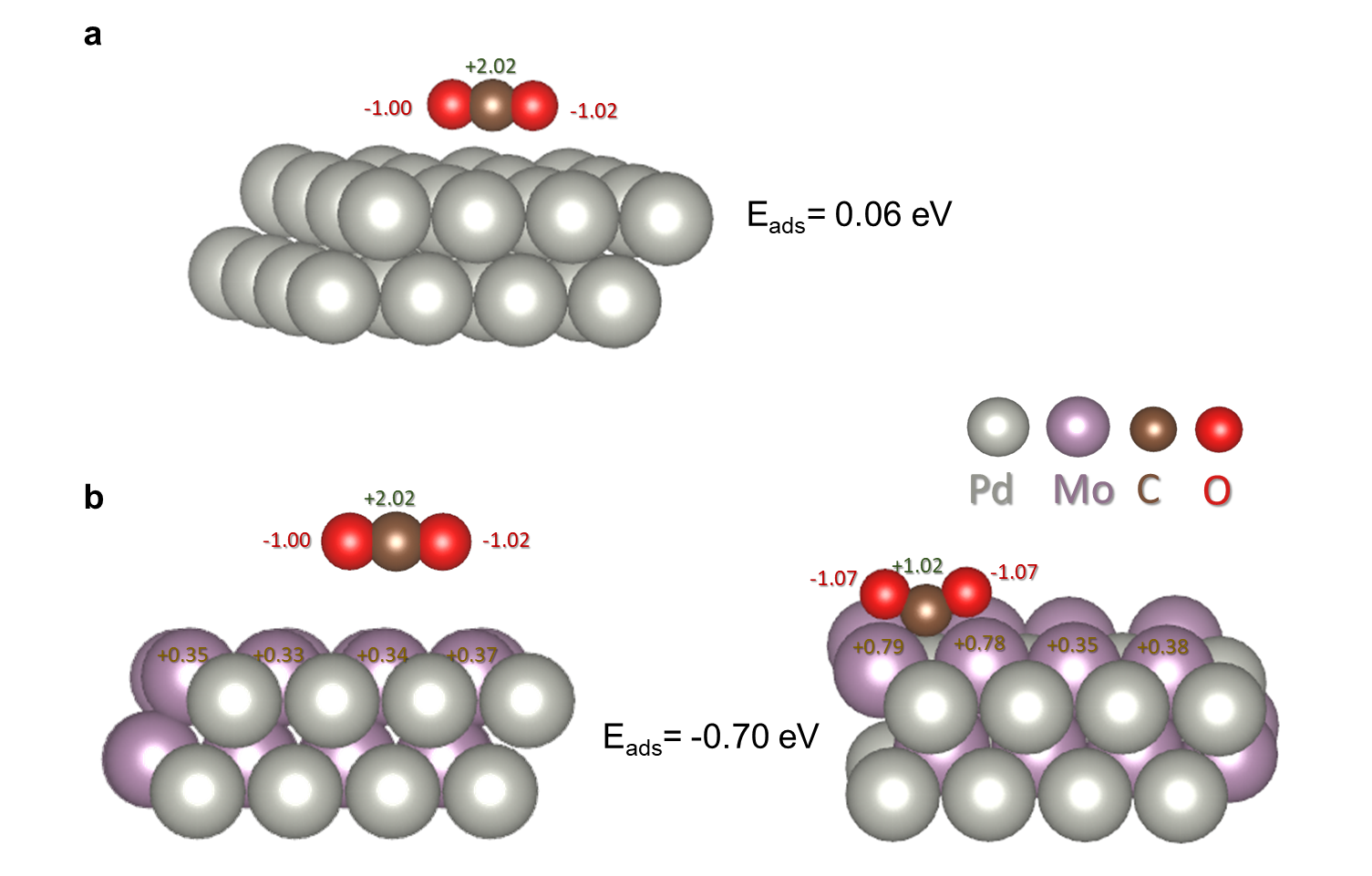


Figure S12. Bader charge analysis results of (a) CO_2_ adsorption energy on Pd(111) surface. (b) gas phase state of CO_2_ and CO_2_ adsorption energy on hcp-PdMo(010) surface and Bader charge analysis results.


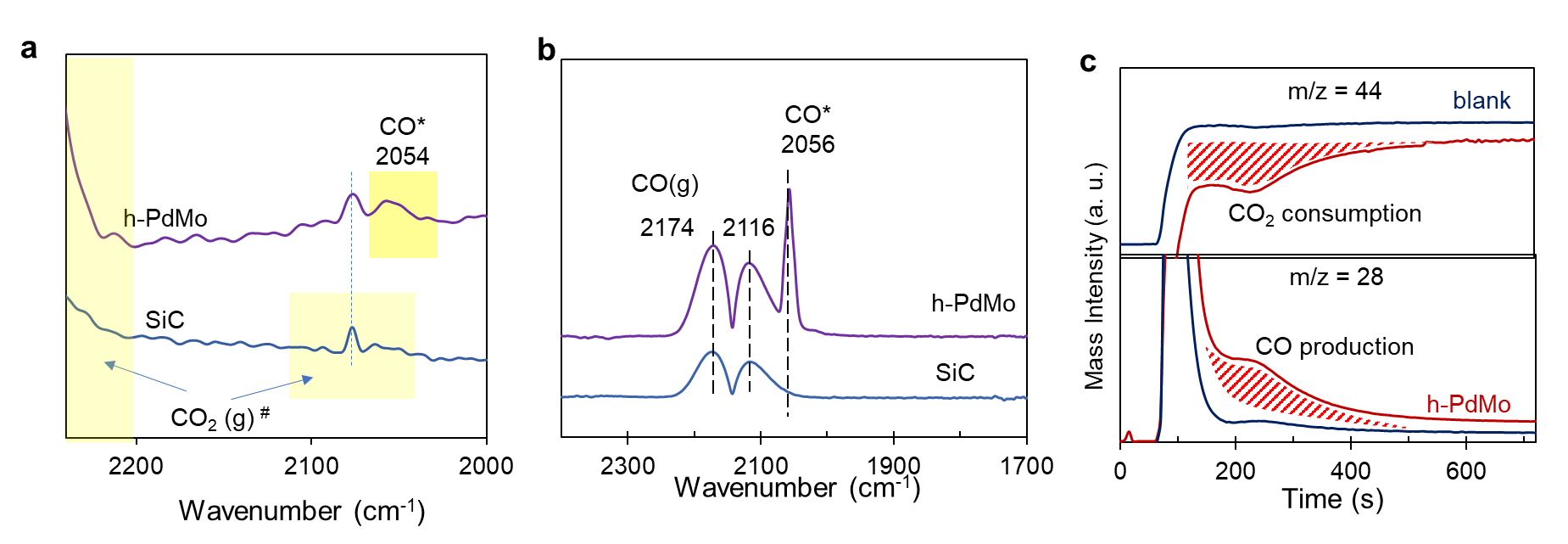


**Figure S13** DRIFT spectra collected over the h-PdMo catalyst and the comparison sample (SiC) under (**a**) a mixed gas (CO_2_:H_2_ = 1:3, 20 mL min^−1^). CO_2_(g)#: related to some gas-phase CO_2_ signals as reported ^[34,35]^ (**b**) 9.85% CO/He (10 mL min^−1^) (**c**) CO_2_-TPSR result of the h-PdMo catalyst. Slanted shades of red area were contributed to the CO_2_ activation and CO production over the h-PdMo catalyst. All experiments were conducted at room temperature.


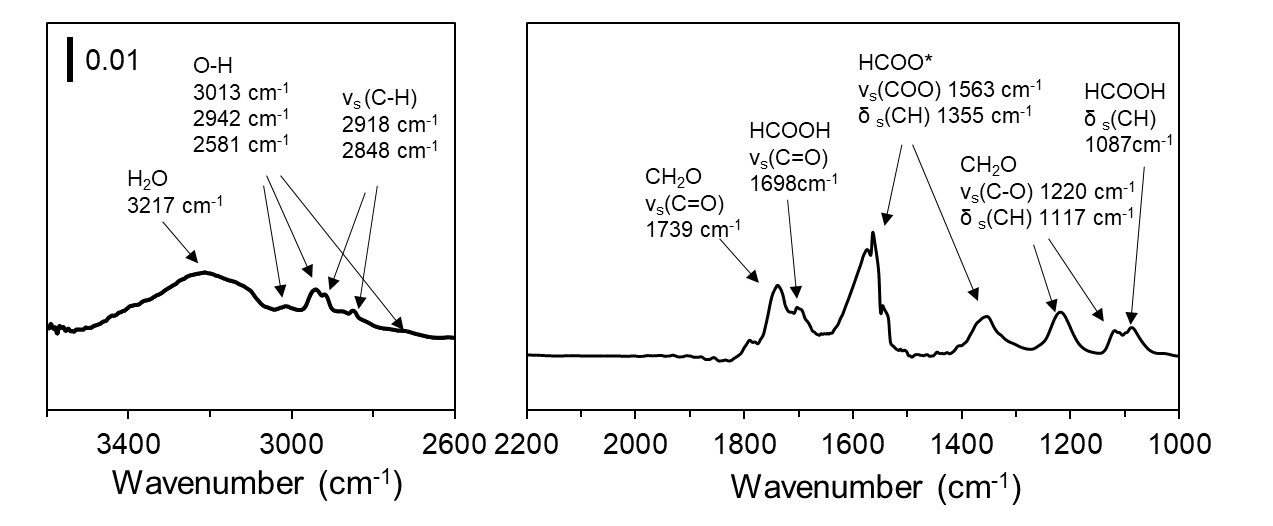


Figure S14. DRIFTS spectra of formic acid adsorption on 5 wt% hcp-PdMo/Mo_2_N catalyst at room temperature. The catalyst was purged with HCOOH (formic acid) at room temperature after the pre-treatment by H_2_ at 300 ^o^C. Then, a vacuum was applied to confirm the adsorption of formic acid.


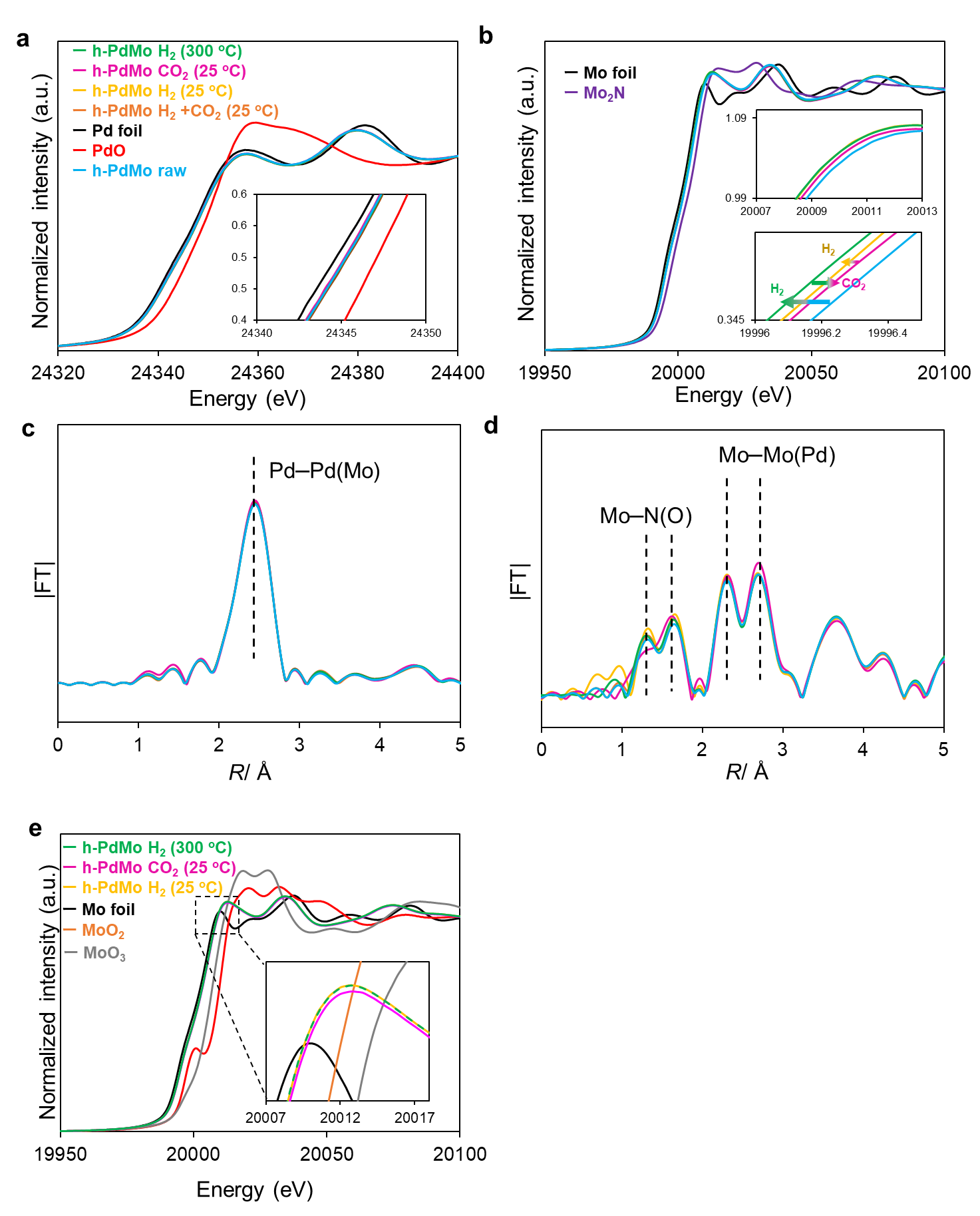


Figure S15. (a) Pd K and (b) Mo K-edge ex situ X-ray absorption near edge structure (XANES) spectra of the hcp-PdMo catalysts and reference compounds. Fourier transform of EXAFS of the hcp-PdMo catalysts, (c) Pd K-edge, (d) Mo K-edge. Air exposed hcp-PdMo catalysts (blue), H_2_ treatment at 300^o^C (green), (e) Mo K-edge ex situ XANES spectra of the hcp-PdMo catalysts and reference compounds. The H₂-treated hcp-PdMo catalysts at 300°C were treated using different gases as follows: CO_2_ treatment at 25^o^C(pink), H_2_ treatment at 25^o^C(yellow), CO_2_ + H_2_ treatment at 25^o^C(orange).


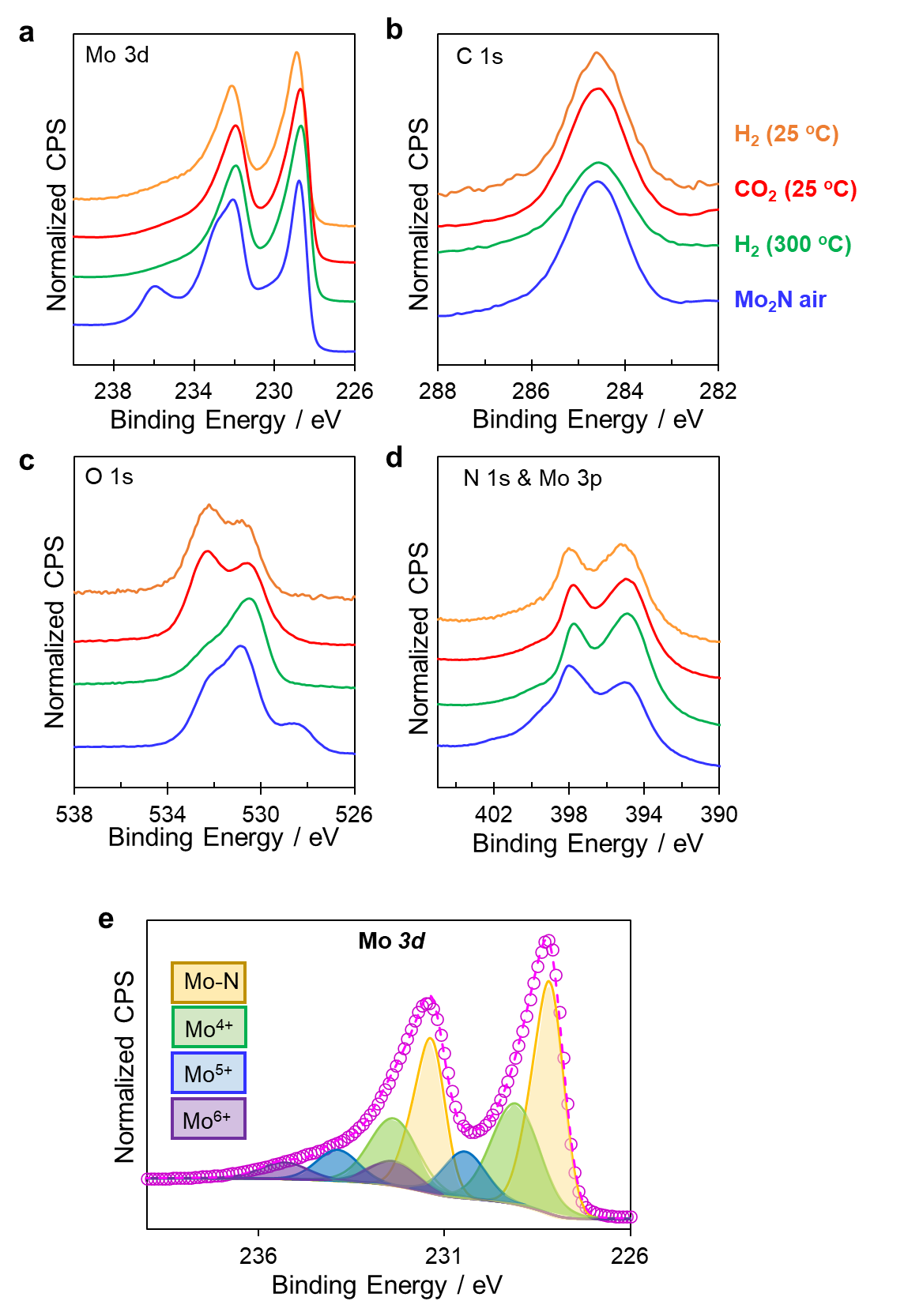


Figure S16. Ex-situ XPS spectra of Mo_2_N catalyst (a) Mo 3d, (b) C 1s (c) O 1s, and (d) N 1s/ Mo 3p. Air exposed Mo_2_N catalysts (blue), H_2_ treatment at 300^o^C (green). The H₂-treated Mo_2_N catalysts at 300°C were treated with CO_2_ at 25^o^C(red), then, the CO₂-treated sample was further treated with H_2_ at 25^o^C(orange). (e). XPS Mo 3d peak fitting spectra of the Mo_2_N catalyst under H_2_ treatment at 300°C.


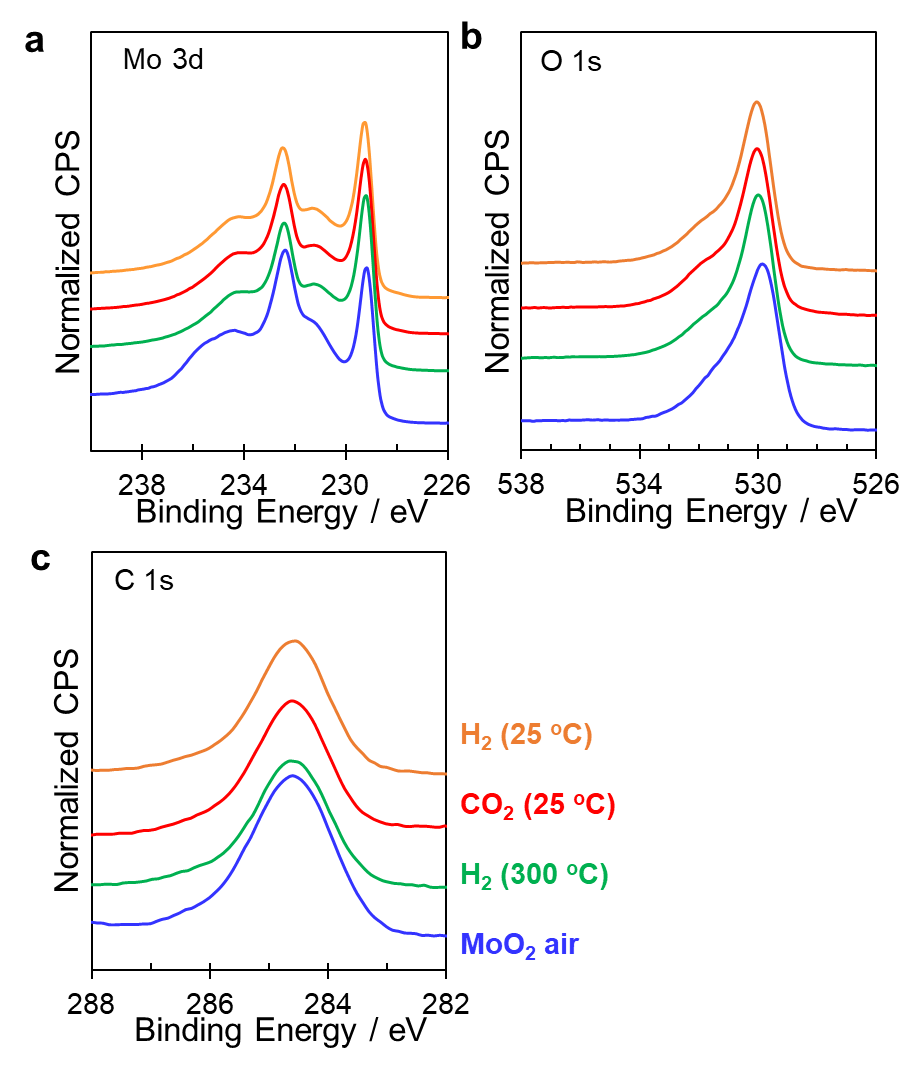


Figure S17. (a) Ex-situ XPS spectra of MoO_2_ catalyst (a) Mo 3d, (b) C 1s, and (c) O 1s. Air exposed MoO_2_ catalysts (blue), H_2_ treatment at 300^o^C (green). The H₂-treated MoO_2_ catalysts at 300°C were treated with CO_2_ at 25^o^C(red), then, the CO₂-treated sample was further treated with H_2_ at 25^o^C(orange).


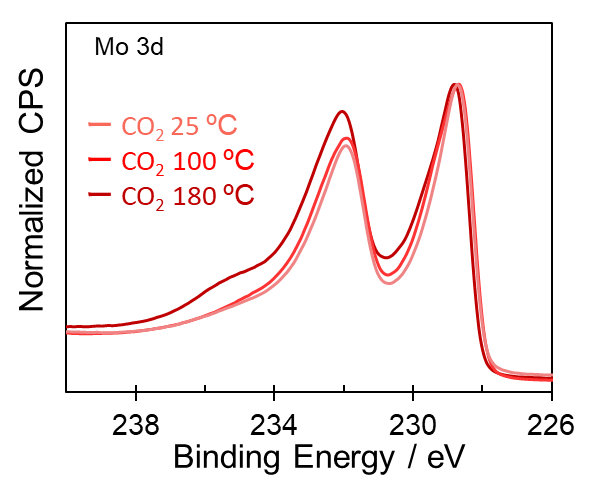


Figure S18. Mo 3d XPS spectra of Mo_2_N treated with CO_2_ gas under different temperature.


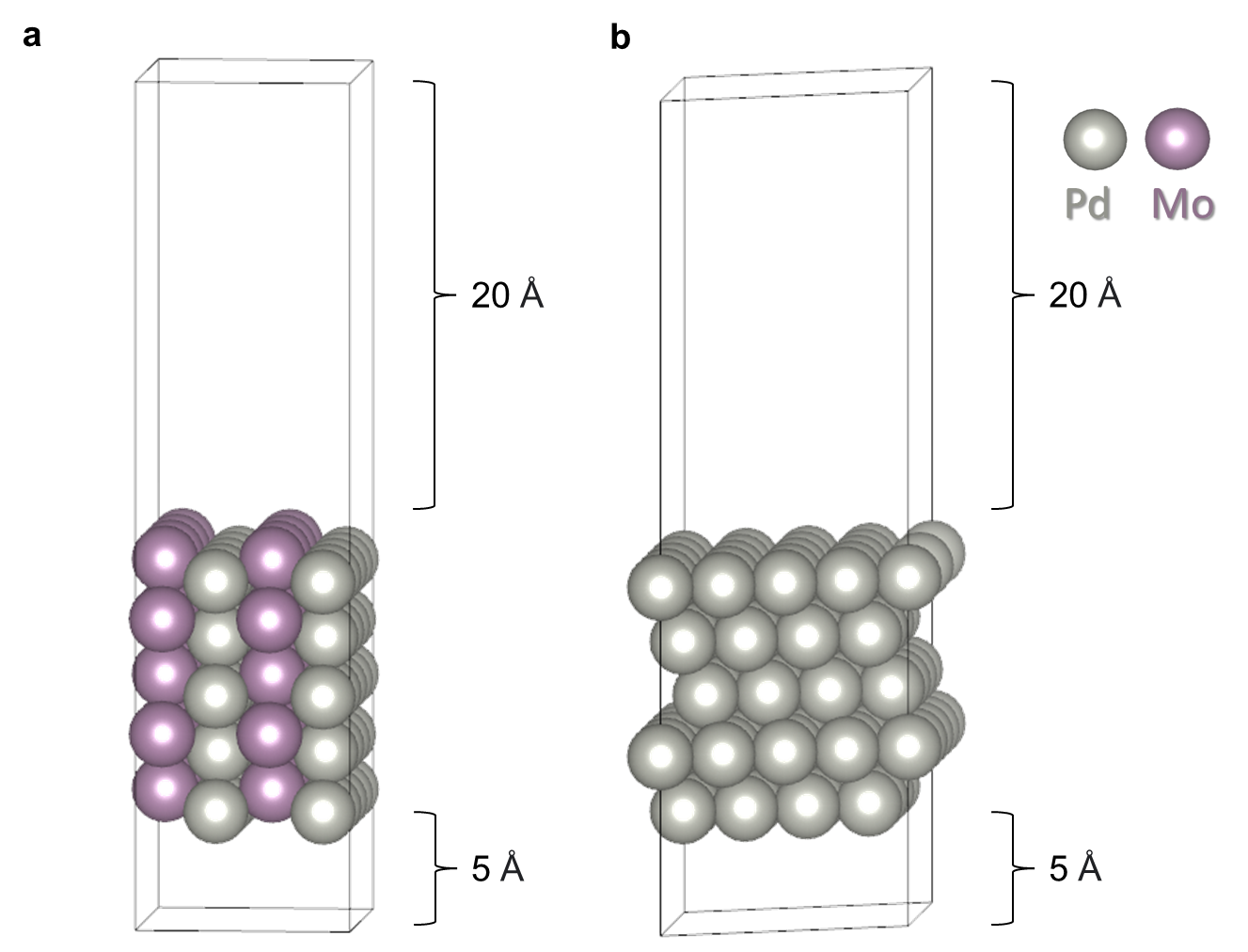


Figure S19. Slab models used in the calculations (**a**) PdMo(010) (**b**) Pd(111).


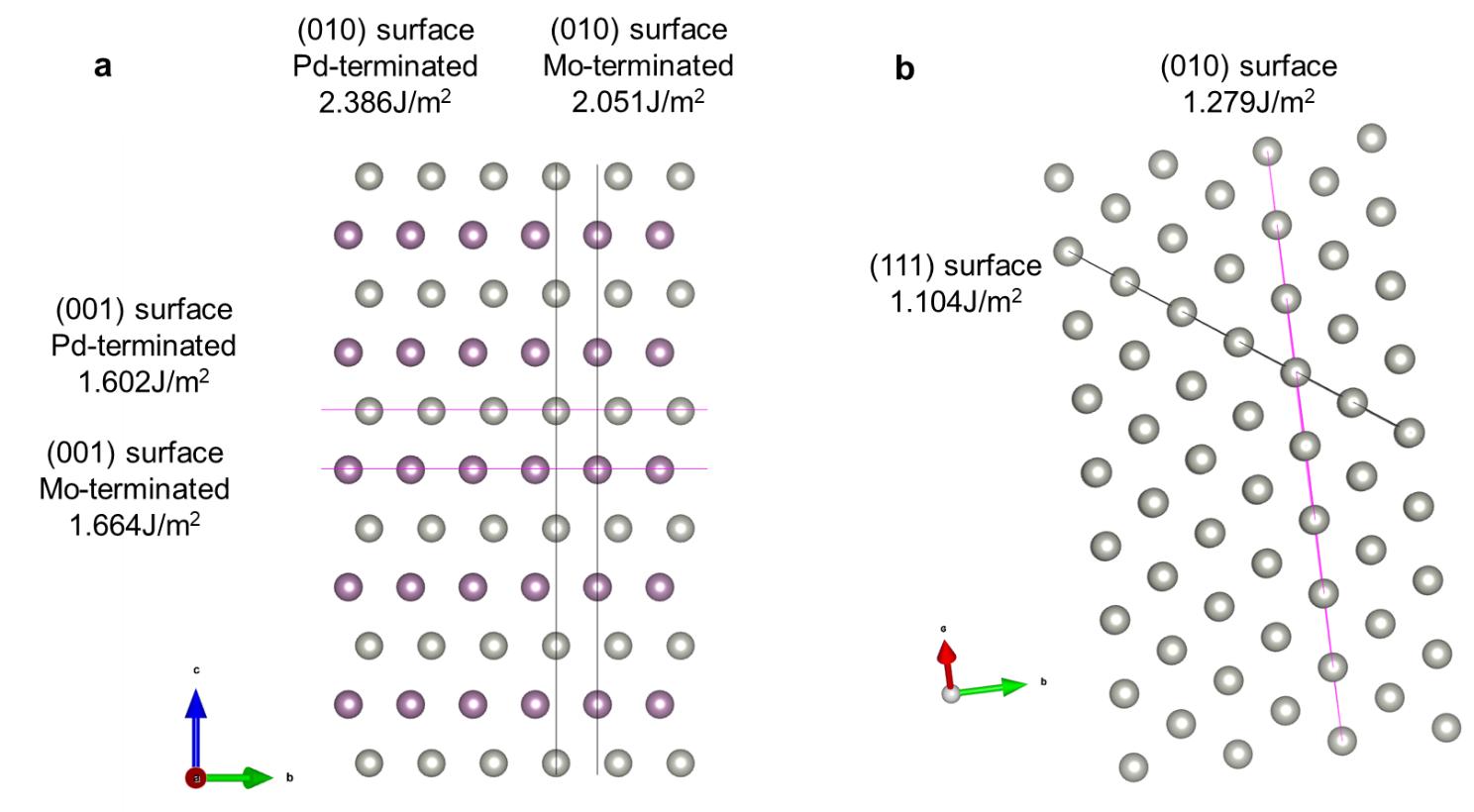


Figure S20. DFT prediction on the cleavage energy for different surfaces of (**a**) h-PdMo and (**b**) Pd.


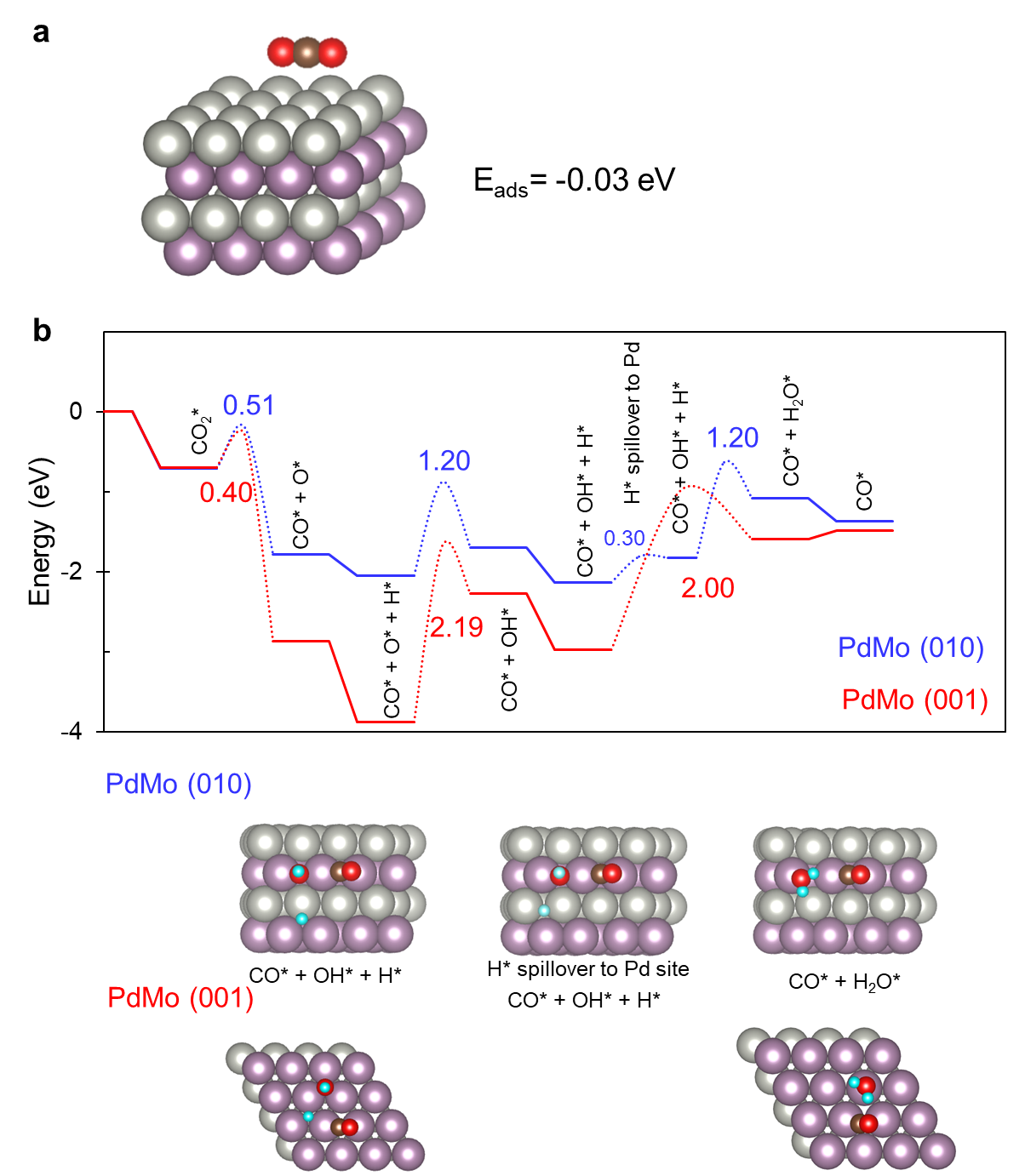
Figure S21. (a) CO_2_ adsorption energy on Pd-terminated (001) surface of PdMo. (b) Energy diagrams for CO_2_ hydrogenation on Mo-terminated PdMo(001) and PdMo(010) surfaces, and the corresponding configurations of optimized intermediates states over two different surfaces.


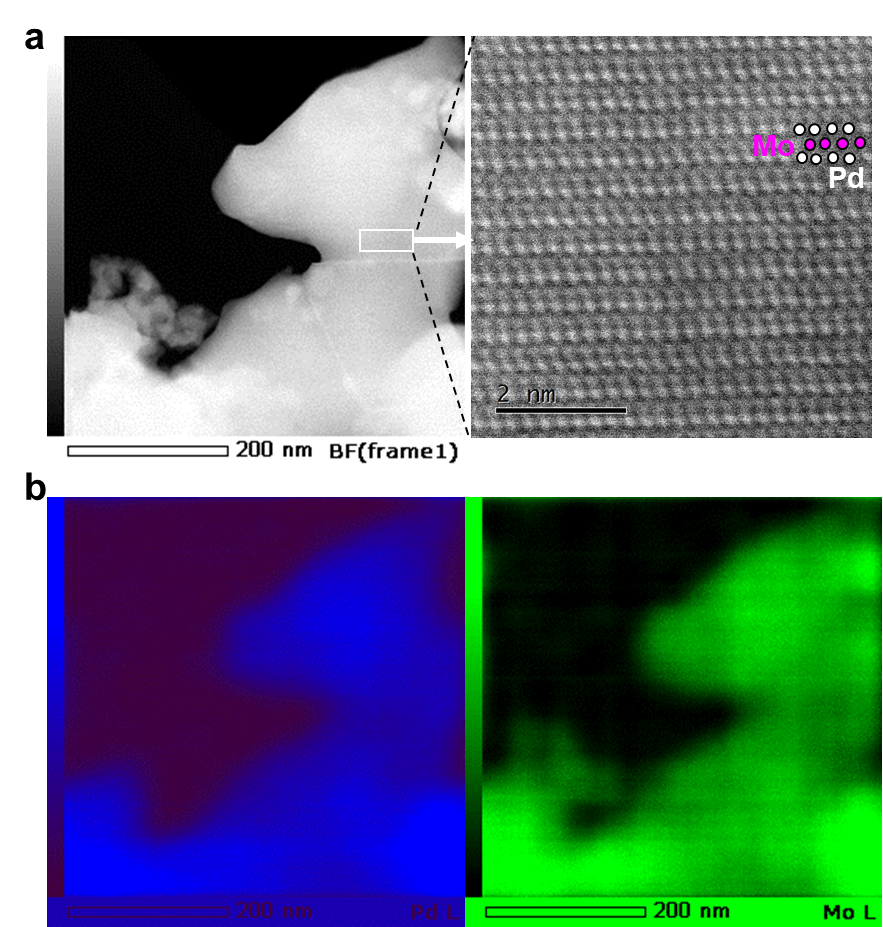


Figure S22. (a) STEM Z-contrast image obtained in HAADF mode (b) EDX mapping image of hcp-PdMo catalysts.


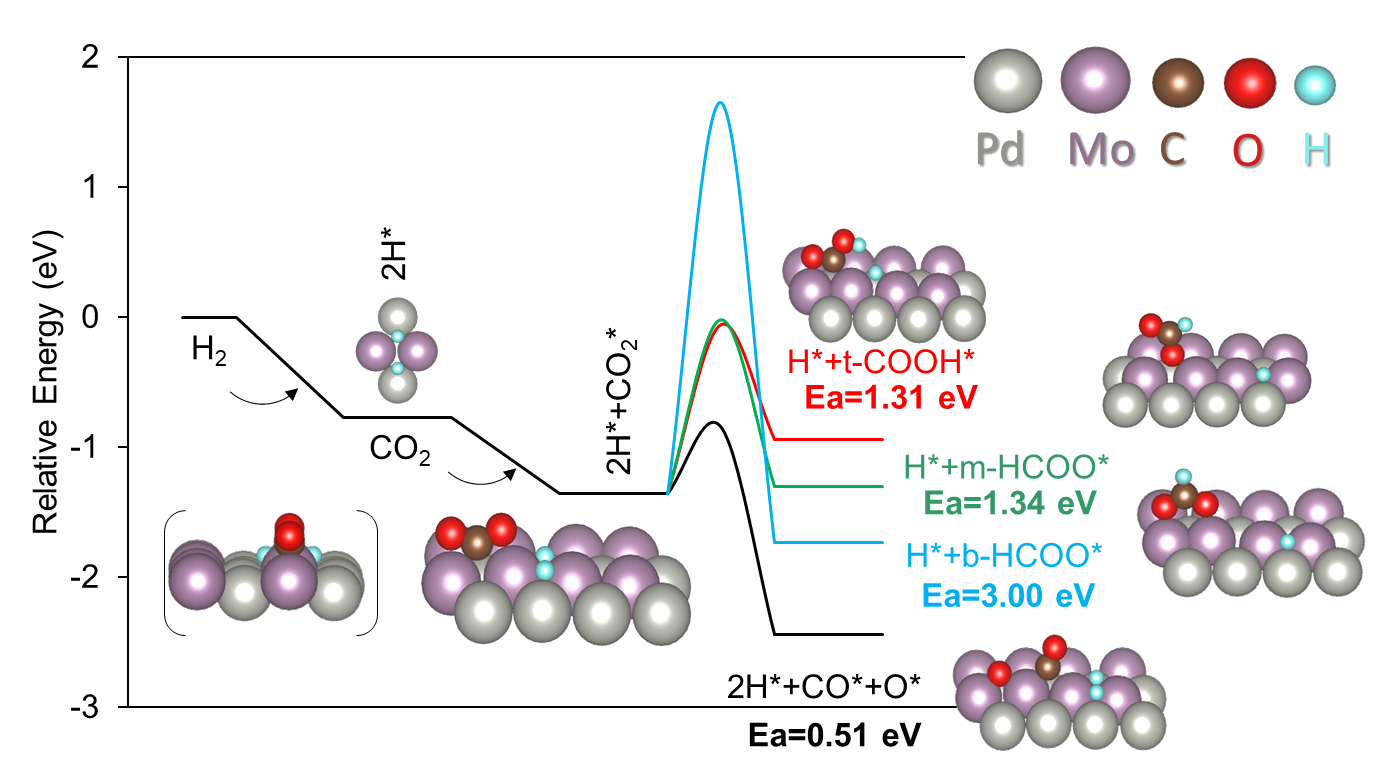


Figure S23. Energy diagram of CO_2_ activation to CO* and hydrogenation to t-COOH*, m-HCOO*, and b-HCOO* over PdMo(010) surface.


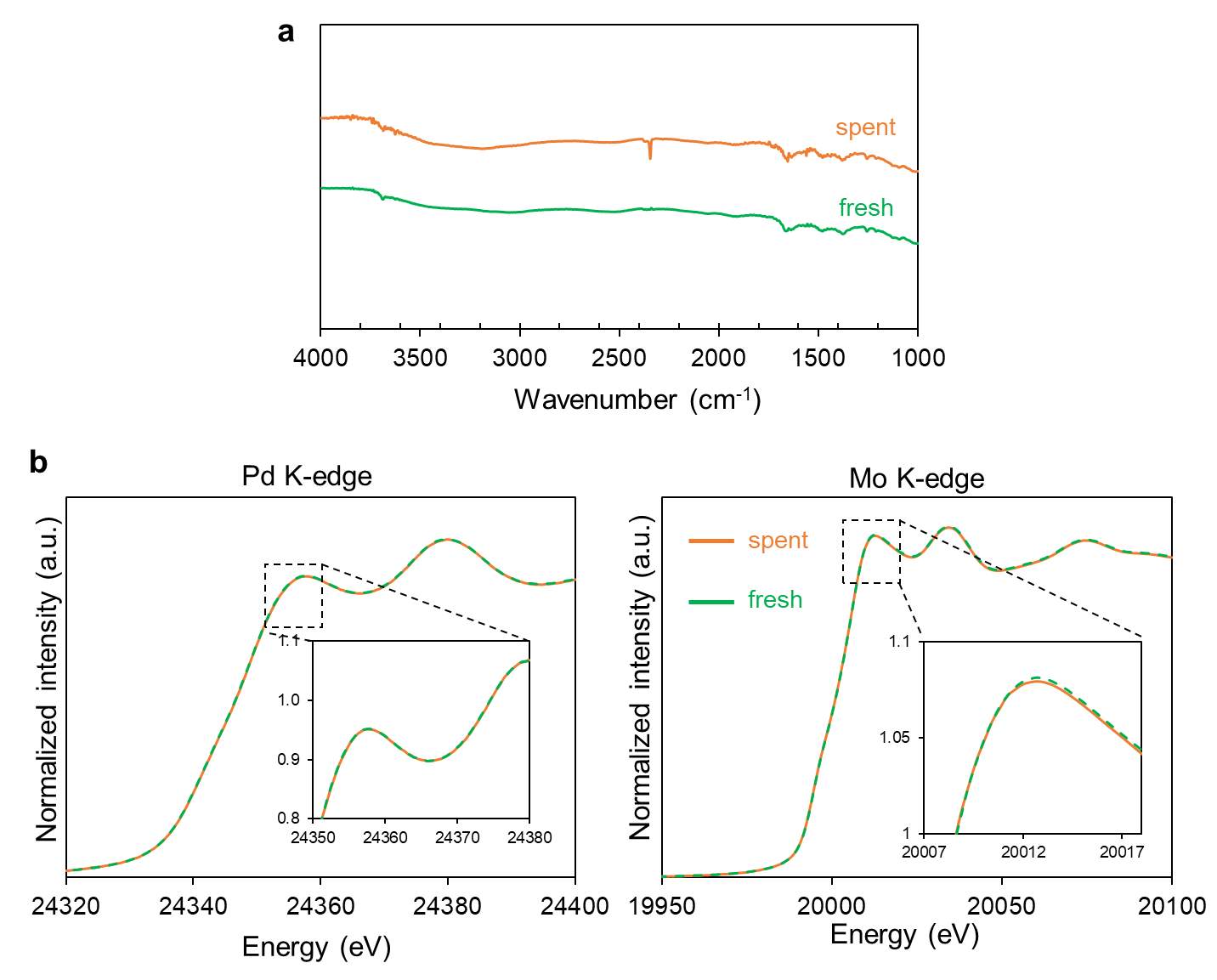


Figure S24 (a) DRIFT spectra (b) Pd K and Mo K-edge XAFS spectra of the fresh and spent h-PdMo catalyst. The spent catalyst is h-PdMo catalyst after reaction at room temperature for 24 h.


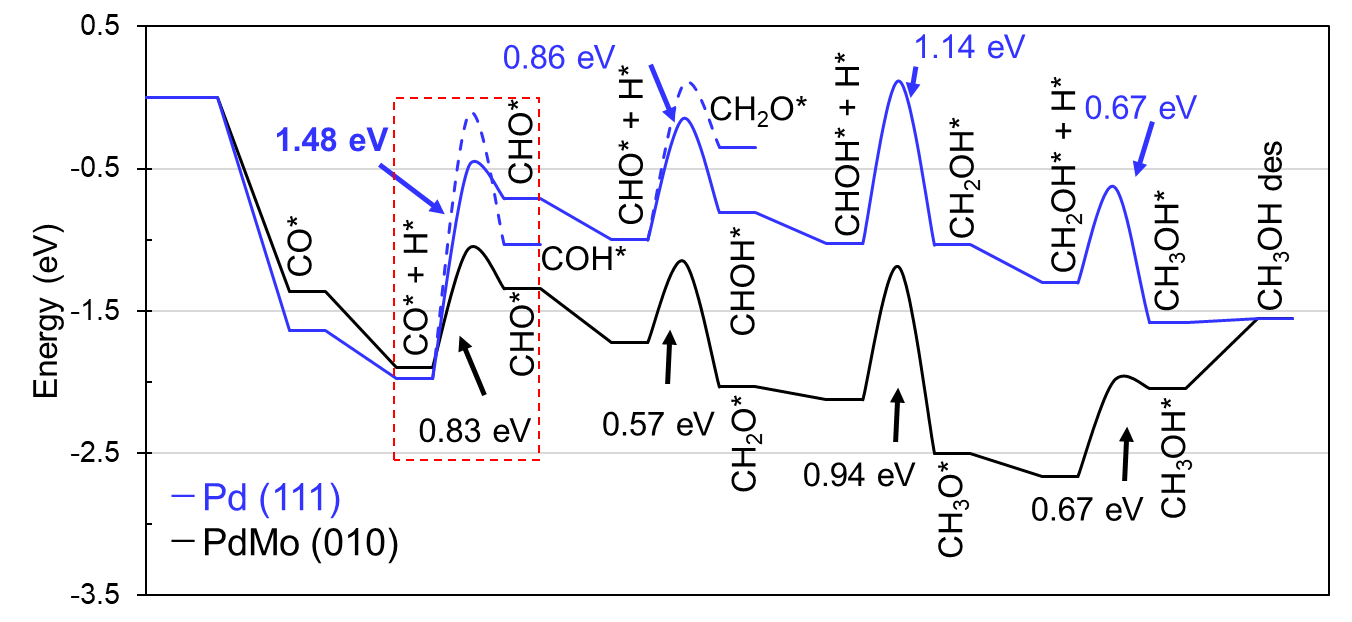


Figure S25. Energy diagrams from CO to CH_3_OH formation on Pd(111) and PdMo(010) surfaces.

Table S1. BET specific surface area of PdMo catalysts calcined at different temperatures.

| Catalyst | S_BET_(m^2^/g) |
| --- | --- |
| 950 °C | <1 |
| 800 °C | 0.74 |
| 750 °C | 2.0 |
| 700 °C | 4.3 |
| 600 °C | 4.7 |
| 500 °C | 5.6 |
| 750 °C (HSA) | 5.6 |

Table S2: Methanol synthesis rate over the PdMo catalyst, commercial CuZnAl catalysts, other reported Mo-based systems, and highly selective methanol synthesis catalysts.

| Catalyst | *T* (°C) | *P* (MPa) | *r*_MeOH_  (*μ*mol g_cat_^-1^ h^-1^) | WHSV  (mL g_cat_^-1^ h^-1^) | CO_2_  conv.(%) | MeOH  selec.(%) |
| --- | --- | --- | --- | --- | --- | --- |
| HSA hcp-PdMo | 25 | 0.9 | 24.6 | 30000 | 0.010 | 100 |
| HSA hcp-PdMo | 25 | 3 | 72.6 | 30000 | 0.021 | 100 |
| HSA hcp-PdMo | 200 | 0.9 | 959 | 30000 | 2.15 | 26 |
| HSA hcp-PdMo | 200 | 3 | 2494 | 30000 | 3.39 | 51 |
| CuZnAl ^This work^ | 80 | 0.9 | 12 | 30000 | 0.004 | 100 |
| CuZnAl ^This work^ | 200 | 3 | 3931 | 30000 | 3.01 | 80 |
| FL-MoS_2_^5^ | 25 | 5 | 15.6 | 1500 | N.D. | N.D. |
| CuZnAl^5^ | 80 | 5 | 10.9 | 1500 | N.D. | N.D. |
| FL-MoS_2_^5^ | 180 | 5 | 4687 | 24000 | 1.7 | 97 |
| CeO_2_ MoP K/SiO_2_^9^ | 230 | 3.1 | 140.4 | 19200 | <2 | 72 |
| CuZnAl^9^ | 230 | 3.1 | 3448 | 19200 | <2 | 76 |
| Cu/Mo_2_CT_x_/SiO_2-6h_^10^ | 230 | 2.5 | 1872 | 60000 | 0.8 | 52 |
| Mo_3_S_4_@NaZSM-5^11^ | 180 | 4 | 1232 | 1200 | 10 | 96 |
| 0.5Ca5Pd5ZnCeO_2_^12^ | 220 | 3 | 1875 | 2400 | 7.7 | 100 |
| Cu/La_2_O_2_CO_3_^13^ | 200 | 3 | 2812 | 12000 | 2.2 | 99.4 |
| Cu-In-Zr-O^14^ | 210 | 2.5 | 93 | 18000 | 0.07 | 100 |
| CuZn@UiO-bpy^15^ | 250 | 4 | 5.4 | 18000 | 3.3 | 100 |
| In_2_O_3_/ZrO_2_^16^ | 200 | 5 | 312 | 20000 | 0.2 | 100 |
| Pd/In_2_O_3_^17^ | 200 | 5 | 1562 | 21000 | 0.5 | 100 |
| ZnO-ZrO_2_^18^ | 220 | 5 | 312 | 24000 | 0.14 | 98.6 |

Table S3. Adsorption wavenumbers of intermediates on hcp-PdMo/Mo_2_N, MoS_2_, and Pd catalyst

|  | hcp-PdMo/Mo_2_N | MoS_2_^5^ | | Pd^6–8^ |
| --- | --- | --- | --- | --- |
| *CO | 2054 | 2078 | 2083-2091 | |
| *ν*(CH) of *CH_3_O | 2852, 2925 | 2846, 2915 | 2860, 2960 | |

Table S4. Summary of composition of each valence of Mo after peak fitting

**
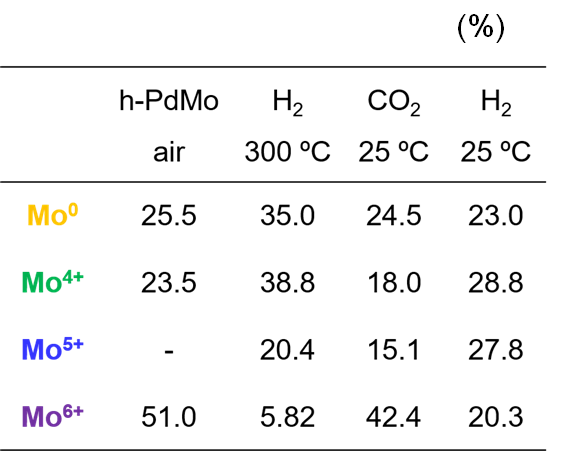
**

**References**

(1) Hammer, B.; Hansen, L. B.; Nørskov, J. K. Improved Adsorption Energetics within Density-Functional Theory Using Revised Perdew-Burke-Ernzerhof Functionals. *Phys Rev B Condens Matter Mater Phys* **1999**, *59* (11), 7413–7421.

(2) Kresse, G.; Furthmü, J. *Efficient Iterative Schemes for Ab Initio Total-Energy Calculations Using a Plane-Wave Basis Set*; **1996**, 54, 11169

(3) Wang, V.; Xu, N.; Liu, J. C.; Tang, G.; Geng, W. T. VASPKIT: A User-Friendly Interface Facilitating High-Throughput Computing and Analysis Using VASP Code. *Comput Phys Commun* **2021**, *267*, 108033.

(4) Momma, K.; Izumi, F. VESTA 3 for Three-Dimensional Visualization of Crystal, Volumetric and Morphology Data. *J Appl Crystallogr* **2011**, *44* (6), 1272–1276.

(5) Hu, J.; Yu, L.; Deng, J.; Wang, Y.; Cheng, K.; Ma, C.; Zhang, Q.; Wen, W.; Yu, S.; Pan, Y.; Yang, J.; Ma, H.; Qi, F.; Wang, Y.; Zheng, Y.; Chen, M.; Huang, R.; Zhang, S.; Zhao, Z.; Mao, J.; Meng, X.; Ji, Q.; Hou, G.; Han, X.; Bao, X.; Wang, Y.; Deng, D. Sulfur Vacancy-Rich MoS_2_ as a Catalyst for the Hydrogenation of CO_2_ to Methanol. *Nat Catal* **2021**, *4* (3), 242–250.

(6) Ebbesen, S. D.; Mojet, B. L.; Lefferts, L. The Influence of Water and PH on Adsorption and Oxidation of CO on Pd/Al_2_O_3_-an Investigation by Attenuated Total Reflection Infrared Spectroscopy. *Phys. Chem. Chem. Phys.* **2009**, *11* (4), 641–649.

(7) Hirano, T.; Kazahaya, Y.; Nakamura, A.; Miyao, T.; Naito, S. Remarkable Effect of Addition of in and Pb on the Reduction of N_2_O by CO over SiO_2_ Supported Pd Catalysts. *Catal Letters* **2007**, *117* (1–2), 73–78.

(8) Ojelade, O. A.; Zaman, S. F. A Review on Pd Based Catalysts for CO_2_ Hydrogenation to Methanol: In-Depth Activity and DRIFTS Mechanistic Study. *Catal. Surv. Asia.* **2020**, *24* (1), 11–37.

(9) Duyar, M. S.; Tsai, C.; Snider, J. L.; Singh, J. A.; Gallo, A.; Yoo, J. S.; Medford, A. J.; Abild‐Pedersen, F.; Studt, F.; Kibsgaard, J.; Bent, S. F.; Nørskov, J. K.; Jaramillo, T. F. A Highly Active Molybdenum Phosphide Catalyst for Methanol Synthesis from CO and CO 2 . *Angew. Chem. Int. Ed.* **2018**, *130* (46), 15265–15270.

(10) Zhou, H.; Chen, Z.; López, A. V.; López, E. D.; Lam, E.; Tsoukalou, A.; Willinger, E.; Kuznetsov, D. A.; Mance, D.; Kierzkowska, A.; Donat, F.; Abdala, P. M.; Comas-Vives, A.; Copéret, C.; Fedorov, A.; Müller, C. R. Engineering the Cu/Mo_2_CTx (MXene) Interface to Drive CO_2_ Hydrogenation to Methanol. *Nat Catal* **2021**, *4* (10), 860–871.

(11) Liu, G.; Liu, P.; Meng, D.; Zhao, T.; Qian, X.; He, Q.; Guo, X.; Qi, J.; Peng, L.; Xue, N.; Zhu, Y.; Ma, J.; Wang, Q.; Liu, X.; Chen, L.; Ding, W. CO_x_ Hydrogenation to Methanol and Other Hydrocarbons under Mild Conditions with Mo_3_S_4_@ZSM-5. *Nat Commun* **2023**, *14* (1), 513.

(12) Malik, A. S.; Zaman, S. F.; Al-Zahrani, A. A.; Daous, M. A.; Driss, H.; Petrov, L. A. Development of Highly Selective PdZn/CeO_2_ and Ca-Doped PdZn/CeO_2_ Catalysts for Methanol Synthesis from CO_2_ Hydrogenation. *Appl Catal A Gen* **2018**, *560*, 42–53.

(13) Chen, K.; Duan, X.; Fang, H.; Liang, X.; Yuan, Y. Selective Hydrogenation of CO_2_ to Methanol Catalyzed by Cu Supported on Rod-like La_2_O_2_CO_3_. *Catal Sci Technol* **2018**, *8* (4), 1062–1069.

(14) Yao, L.; Shen, X.; Pan, Y.; Peng, Z. Synergy between Active Sites of Cu-In-Zr-O Catalyst in CO_2_ Hydrogenation to Methanol. *J Catal* **2019**, *372*, 74–85.

(15) An, B.; Zhang, J.; Cheng, K.; Ji, P.; Wang, C.; Lin, W. Confinement of Ultrasmall Cu/ZnO_2_ Nanoparticles in Metal-Organic Frameworks for Selective Methanol Synthesis from Catalytic Hydrogenation of CO_2_. *J Am Chem Soc* **2017**, *139* (10), 3834–3840.

(16) Martin, O.; Martín, A. J.; Mondelli, C.; Mitchell, S.; Segawa, T. F.; Hauert, R.; Drouilly, C.; Curulla‐Ferré, D.; Pérez‐Ramírez, J. Indium Oxide as a Superior Catalyst for Methanol Synthesis by CO_2_ Hydrogenation. *Angew. Chem. Int. Ed.* **2016**, *128* (21), 6369–6373.

(17) Rui, N.; Wang, Z.; Sun, K.; Ye, J.; Ge, Q.; Liu, C. jun. CO_2_ Hydrogenation to Methanol over Pd/In_2_O_3_: Effects of Pd and Oxygen Vacancy. *Appl Catal B* **2017**, *218*, 488–497.

(18) Wang, J.; Li, G.; Li, Z.; Tang, C.; Feng, Z.; An, H.; Liu, H.; Liu, T.; Li, C. A Highly Selective and Stable ZnO-ZrO_2_ Solid Solution Catalyst for CO_2_ Hydrogenation to Methanol. *Sci. Adv.* **2017**, *3*, e1701290.
